# Il-6 signaling between ductal carcinoma in situ cells and carcinoma-associated fibroblasts mediates tumor cell growth and migration

**DOI:** 10.1186/s12885-015-1576-3

**Published:** 2015-08-13

**Authors:** Kingsley O. Osuala, Mansoureh Sameni, Seema Shah, Neha Aggarwal, Michelle L. Simonait, Omar E. Franco, Yan Hong, Simon W. Hayward, Fariba Behbod, Raymond R. Mattingly, Bonnie F. Sloane

**Affiliations:** 1 Department of Pharmacology, Wayne State University, 540 East Canfield, Detroit, MI 48201 USA; 2 Cancer Biology Program, Wayne State University, 540 East Canfield, Detroit, MI 48201 USA; 3 Department of Physiology, Wayne State University, 540 East Canfield, Detroit, MI 48201 USA; 4 School of Medicine, Wayne State University, 540 East Canfield, Detroit, MI 48201 USA; 5 Department of Surgery, NorthShore University Health System Research Institute, 1001 University Place, Evanston, IL 60201 USA; 6 Division of Cancer and Developmental Biology, The University of Kansas Medical Center, 3901 Rainbow Boulevard, Kansas City, KS 66160 USA

**Keywords:** Ductal carcinoma in situ (DCIS), Carcinoma-associated fibroblasts (CAFs), Interleukin-6 (IL-6), Breast cancer, 3D cell culture

## Abstract

**Background:**

Ductal carcinoma in situ (DCIS) is a non-obligate precursor lesion of invasive breast cancer in which approximately half the patients will progress to invasive cancer. Gaining a better understanding of DCIS progression may reduce overtreatment of patients. Expression of the pro-inflammatory cytokine interleukin-6 increases with pathological stage and grade, and is associated with poorer prognosis in breast cancer patients. Carcinoma associated fibroblasts (CAFs), which are present in the stroma of DCIS patients are known to secrete pro-inflammatory cytokines and promote tumor progression.

**Methods:**

We hypothesized that IL-6 paracrine signaling between DCIS cells and CAFs mediates DCIS proliferation and migration. To test this hypothesis, we utilized the mammary architecture and microenvironment engineering or MAME model to study the interactions between human breast CAFs and human DCIS cells in 3D over time. We specifically inhibited autocrine and paracrine IL-6 signaling to determine its contribution to early stage tumor progression.

**Results:**

Here, DCIS cells formed multicellular structures that exhibited increased proliferation and migration when cultured with CAFs. Treatment with an IL-6 neutralizing antibody inhibited growth and migration of the multicellular structures. Moreover, selective knockdown of IL-6 in CAFs, but not in DCIS cells, abrogated the migratory phenotype.

**Conclusion:**

Our results suggest that paracrine IL-6 signaling between preinvasive DCIS cells and stromal CAFs represent an important factor in the initiation of DCIS progression to invasive breast carcinoma.

**Electronic supplementary material:**

The online version of this article (doi:10.1186/s12885-015-1576-3) contains supplementary material, which is available to authorized users.

## Background

Ductal carcinoma in situ (DCIS) of the breast is a pre-invasive lesion and a risk factor for subsequent invasive ductal carcinoma (IDC) [1]. DCIS represents about 20 % of newly diagnosed breast cancers in the United States [2]. If left untreated, approximately half of DCIS tumors will progress to IDC while half will remain indolent [3, 4]. Although there are many subtypes of DCIS, it is not currently possible to identify which will progress. This has led to aggressive treatments, specifically radiation with either lumpectomy or mastectomy [5].

Components of the tumor microenvironment are increasingly implicated in the progression of many cancers. Early morphological and physiological changes in breast epithelium are minimal, and compounding factors such as tumor-suppressive paracrine signaling from myoepithelial cells [6] or the extracellular matrix [7] may hide early indicators of ductal cell aberration. Such changes may include, but are not limited to: gene expression modulation, epigenetic alterations, and loss of genomic stability in both the epithelial and stromal compartments. In the tumor microenvironment, carcinoma-associated fibroblasts (CAFs) represent a fibroblast population or mixture of sub-populations that can promote tumor progression [8–13]. Although this mechanism is not fully understood, it is known that CAFs secrete numerous cytokines and growth factors [14].

Interleukin 6 (IL-6) is a pro-inflammatory cytokine shown to alter cell morphology, modulate cell migration and the epithelial to mesenchymal transition [15–17]. Many of these processes occur upon IL-6 activation of the transmembrane IL-6 receptor (IL-6R), which heterodimerizes with the ubiquitously expressed cell surface receptor glycoprotein 130 (gp130). Downstream activation of the Janus kinase/signal transducers and activators of transcription (JAK/STAT) pathway initiates IL-6 target gene transcription [18, 19]. Alternatively, secreted IL-6 can bind to the soluble IL-6 receptor (sIL-6R), which then binds gp130 at the cell surface and initiates intracellular signaling. This form of IL-6 signaling has been coined “IL-6 trans-signaling” (IL-6TS) [20]. IL-6 has been linked to the upregulation of proteases such as cysteine cathepsins and matrix metalloproteinases that are known to play a role in cancer progression [21, 22]. The IL-6 signaling axis is commonly upregulated in invasive cancers, suggesting that IL-6 may be an important mediator of events involved in tumor cell invasion [23–25].

The effects of IL-6 signaling inhibitors on breast cancer cell morphology and proliferation have been evaluated in monotypic cultures. Two studies have shown that inhibiting autocrine IL-6 signaling in either triple negative breast cancer cell lines [26], or the ER-positive MCF7 cell line [27] significantly inhibited cell growth. Additionally, Leslie et al. show that knockdown of IL-6 in an invasive variant of the MCF10A cells, MCF10A-H-RasV12, inhibited cell migration in a transwell assay, and inhibited growth in a xenograft mouse model [28]. Although these studies evaluated paracrine signaling, cells were treated with exogenous recombinant protein rather than co-culturing different cell types. Therefore the authors were unable to evaluate in 3D the dynamic cell:cell interactions between two separate human cell types or the cell:microenvironment interactions.

Our 3D mammary architecture and microenvironment engineering (MAME) culture model mimics in vivo architecture, providing a suitable setting to study cell:cell interactions and, notably, physiologically relevant cell:cell signaling over time. Additionally, 3D in vitro cell culture models better represent in vivo tumor drug response, which would facilitate efficacious therapy development at the preclinical stage [29]. Here we examine the role of IL-6 in progression of pre-invasive breast DCIS to an invasive phenotype, and show how co-culture of DCIS cells with CAFs changes DCIS growth and invasive potential.

## Methods

### Cell lines

MCF10A human breast non-transformed epithelial cells, MCF10.DCIS and SUM102 human breast DCIS cells, and WS-12Ti human breast carcinoma-associated fibroblasts were provided by Dr. Bonnie F. Sloane. All primary fibroblasts were derived from human breast tissue. CAF40T were derived from biopsy tissue diagnosed as invasive carcinoma. NAF98 were derived from benign tissue. Both CAF40T and NAF98 fibroblast cell lines were provided by Dr. Simon W. Hayward. These fibroblasts were immortalized in Dr. Sloane’s lab and are designated CAF40TKi and NAF98i, respectively. The FB-NF, NAF-FB, and FB-CAF primary fibroblasts were derived from patient biopsies diagnosed as: benign (FB-NF, NAF-FB), or invasive carcinoma with accompanying DCIS (FB-CAF) and provided by Dr. Fariba Behbod. The FB-NF fibroblasts were immortalized in Dr. Sloane’s lab and designated FB-NF-i. The FB-CAF and NAF-FB fibroblasts were not immortalized. All patient derived cells were received de-identified and therefore are exempt from IRB oversight.

### Cell culture

In this study we utilized non-tumor forming MCF10A human breast epithelial cells [30] and the human DCIS cell lines MCF10.DCIS and SUM102, which were maintained as previously described [31]. See supplemental methods for more detailed information (Additional file 1). All 3D MAME cultures were performed using Cultrex (3433-005-01, Trevigen, Gaithersburg, MD) similar to previously described [32]. Briefly, cell culture dishes were coated with 100 % Cultrex. Cells were added on top of solidified Cultrex and allowed to adhere for 30–45 min before being overlaid with 2 % Cultrex in phenol red-free DMEM F12 media containing 2.5 % fetal bovine serum (Additional file 2: Figure S1). In co-culture experiments, fibroblasts were added first and allowed to adhere before adding tumor cells. Once tumor cells had adhered to the matrix, an overlay of 2 % Cultrex was added. Media were changed every 4 days.

### Measurement of multicellular structures

Differential interference contrast (DIC) images of three random fields at 20X magnification were used to measure multicellular structures. Three individuals of whom two were study-blinded measured the diameter and perimeter of structures and the number and length of interconnections between structures. All three data sets were used in the quantification. This analysis was performed using Zen imaging software (Zeiss, Thornwood, NY). Volume measurements were obtained using 3D fluorescent images and quantified using Volocity software (Perkin-Elmer, Waltham, Mass).

### Gene expression

RNA was isolated from cells grown in either 2D monolayer or 3D MAME cultures. For 2D culture, cells were washed in phosphate buffered saline and harvested using 0.5 % Trypsin/EDTA (Life Technologies, Foster City, CA) and pelleted. The cell pellets were resuspended in TRIzol® Reagent (Life Technologies, Foster City, CA) for RNA extraction. All qRT-PCR reactions were performed using Taqman Assays (Life Technologies, Foster City, CA). See supplemental list of Taqman Assays (Additional file 3: Table S1).

### ELISA

ELISA kits (human IL6R-ab46029, human IL6-ab46044, and human GAPDH-ab119627) were purchased from Abcam® (San Francisco, CA). Aliquots of lysates were collected for ELISA assays and measurement of total DNA.

### Immunohistochemistry

A breast tissue microarray (BR8011) was purchased from US Biomax® (Rockville, MD). Dr. Fariba Behbod provided patient tissue microarrays, and biopsy sections were purchased from ProSci Incorporated (cat # 10–010 and 10–003, Poway, CA). The thickness of all tissue sections immunostained were 10-microns.

### Immunofluorescence

Nuclei were labeled with Hoechst (33342, Thermo Scientific) or EDU (Life Technologies, Foster City, CA). Polyclonal antibodies to human IL-6 (AF-206-NA, R&D Systems, Minneapolis, MN) were used at a concentration of 1 μg/ml. Mono-specific antibodies to human cathepsin B have been previously isolated and characterized [33]. Cathepsin B immunostaining was performed as previously described with the exception that 1 % Tween 20 replaced the 0.01 % saponin [34]. For some studies, CAF40TKi were pre-labeled, prior to seeding in MAME co-cultures, utilizing CellTrace CFSE (carboxyfluorescein diacetate succinimidyl ester; Life Technologies, Foster City, CA) according to the manufacturer’s protocol.

### Drug treatments

For treatment of MAME cultures with IL-6 nAb (R&D Systems, AF-206-NA), we added 1 μg/ml of IL-6 nAb in the 2 % Cultrex overlay to 3D cultures on the first day of culture and refreshed with IL-6 nAb and 2 % overlay every 4 days. The antibody concentration was selected based on preliminary studies in which we determined the lowest concentration needed to inhibit growth of tumor structures. Oxymatrine (Sigma, St. Louis, MO) at 1-mg/ml (3.7 mM) was added 24 hours after cell seeding and was replaced with fresh drug every 4 days. The oxymatrine concentration was determined empirically based on the concentration at which proliferation was inhibited to 50 % of control. The protease inhibitors CA074Me and E64d (Sigma, St. Louis, MO) were used at a concentration of 10 μM [35].

### Live cell proteolysis assay

Dye-quenched collagen IV (DQ-collagen IV, Life Technologies, Foster City, CA) was used admixed in Cultrex as previously described [32]. MAME cultures in optical glass bottom cell culture dishes were imaged live for a period of 10 to 60 min under 5 % CO_2_ at 37 °C.

### Confocal microscopy

Confocal microscopy was performed on either a Zeiss LSM 510 or LSM 780 upright confocal microscope (Zeiss, Thornwood, NY). All cell cultures used for imaging were seeded on 40 mm optical glass bottom culture dishes at a density of 45 cells/mm^2^ (~5000 cells/dish).

### Statistics

Data were statistically analyzed using Student’s *t*-test on GraphPad Prism 6.0 (GraphPad Software Inc., La Jolla, CA).

### Ethics statement

All human subject materials and experiments in this study have been reviewed by the Wayne State University Institutional Review Board and deemed exempt according to the definition codified in the common rule at 45 CFR 46.102(d)(f).

## Results

### Human breast DCIS cells express pro-inflammatory cytokines

**Fig. 1:**
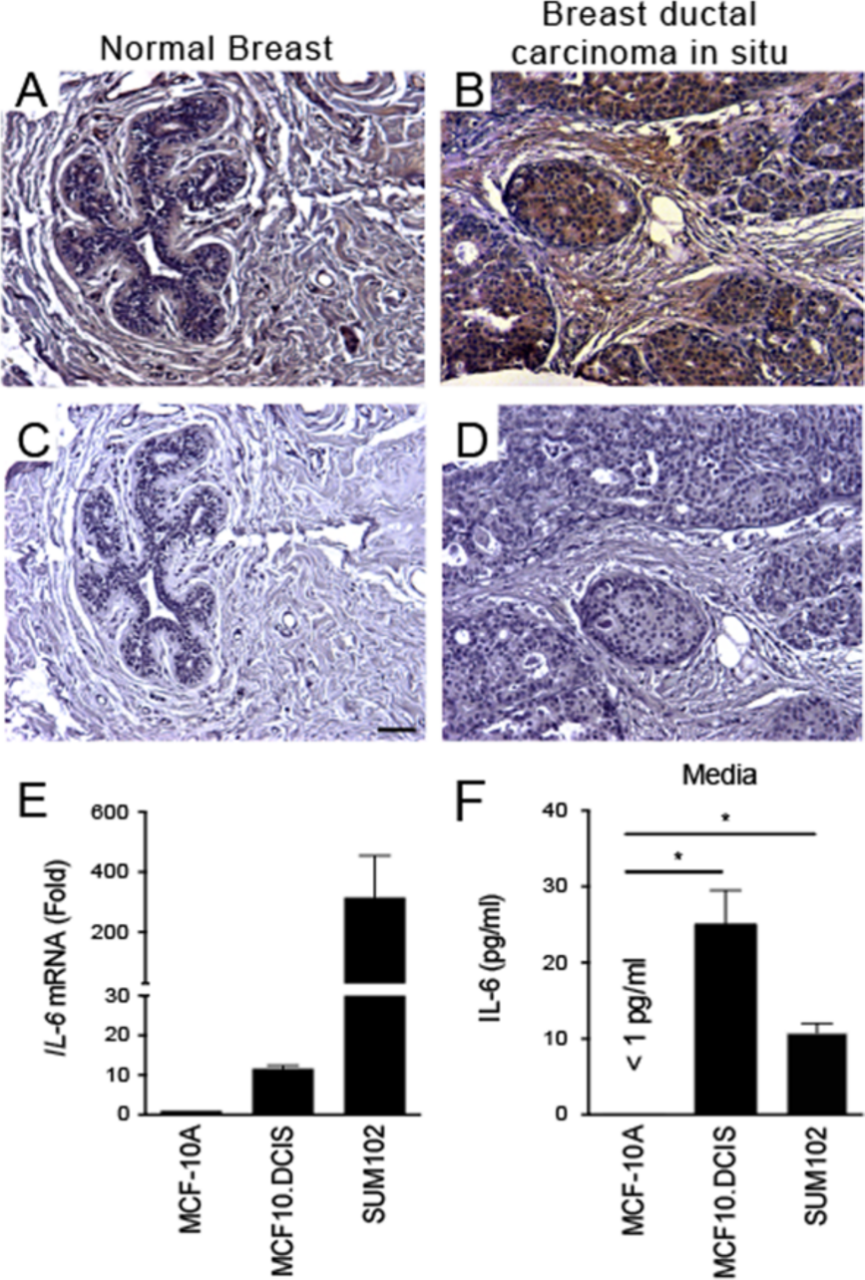
Breast DCIS cells overexpress proinflammatory markers. Representative images of immunohistochemistry targeting IL-6 protein in normal breast tissue (**a**), and breast DCIS (**b**) (N = 61). **c** and **d** Hematoxylin staining of serial sections from tissue shown in panel A and B. All images are 20X magnification. Scale bar equals 100 μm. **e** Evaluation of *IL-6* gene expression in DCIS cells via qRT-PCR; the isogenic MCF10.DCIS cells and the non-isogenic SUM102 cell line were analyzed against the non-transformed MCF10A cell line (N = 3). **f** Secretion of IL-6 protein from DCIS cell lines and non-transformed MCF10A cells as determined by ELISA. *P < 0.05, Student’s *t*-test; mean ± SD

Increased levels of pro-inflammatory cytokines, including IL-6, in tumors and serum of breast cancer patients correlate with poor prognosis [26, 36–38]. Immunohistochemistry on breast tissue from female patients, age 30 to 81 with an average age of 49.5, confirmed IL-6 protein expression in 65 % of patient samples diagnosed with DCIS (Fig. 1a and b, cf. 1c and d).

We utilized a 3D MAME culture model (Additional file 2: Figure S1), to study the role of IL-6 signaling between human breast DCIS cells and human breast CAFs. The MAME model allows for co-culture of multiple cell types in the context of a three dimensional microenvironment. Our model is advantageous over commercially available 3D platforms as it can be utilized for live cell microscopy, and imaging live cell functional assays (such as proteolysis) [32]. MAME cultures are tractable and can be altered to grow multiple cell types at various ratios or at various relative positions within the matrix. Additionally, the reduced density overlay allows for real-time collection and analysis of cytokine secretion without disrupting the longitudinal growth of the culture.

Here we examined the expression of *IL-6* and the associated pro-inflammatory genes *interleukin 1β (IL1β)* and *nuclear factor kappa light chain enhancer of activated B cells 1* (*NFκB1*) in human breast MCF10.DCIS and SUM102 cell lines. Our data show higher IL-6 mRNA levels in both MCF10.DCIS and SUM102 cell lines, as compared to the MCF10A breast epithelial cell line (Fig. 1e). Levels of IL-6 protein in lysates of the three cell lines were below the level of detection. When we measured secreted IL-6 in conditioned media from MCF10.DCIS or SUM102 cultures, we found that both DCIS cell lines secreted 10 to 30-fold more IL-6 than the MCF10A cells (Fig. 1f).

### Blocking IL-6 autocrine signaling inhibits MCF10.DCIS growth

**Fig. 2:**
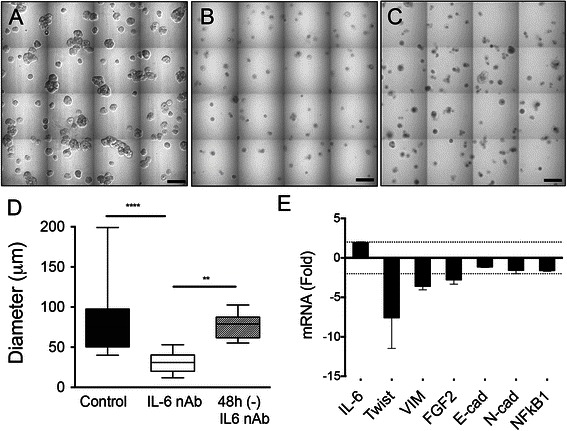
IL-6 neutralizing antibody (nAb) inhibits growth of MCF10.DCIS structures. Representative contiguous 16-tiled DIC images of MCF10.DCIS cells grown in MAME cultures for 8 days. MAME culture of MCF10.DCIS cells treated with an isotype-matched antibody against IgG (control) (8 days) (**a**), or 1 μg /ml IL-6 nAb (**b**) (8 days). MCF10.DCIS cells treated with IL-6 nAb followed by a 48 h treatment-free recovery period prior to imaging on day 10 (**c**). N = 3, Scale bars, 200 μm. **d** Diameter of MCF10.DCIS structures in the presence of control antibody, IL-6 nAb, or 48 h recovery from IL-6 nAb. N = 20-40 measurements /tiled DIC image (N = 3). ****P ≤ 0.0001, **P ≤ 0.01. **e** Evaluation of gene expression of invasive tumor cell markers in MAME cultures treated with IL-6 nAb (N = 3). Fold difference as compared to control cultures. Dashed line indicates 2-fold threshold. Student’s *t*-test; mean ± SD

To test whether blocking IL-6 autocrine signaling affected DCIS cell growth, we treated MCF10.DCIS MAME cultures for 8 days with either an IL-6 neutralizing antibody (nAb) or an equivalent concentration of a species and isotype matched antibody (Fig. 2). IL-6 nAb treatment resulted in a reduction in diameter of the multicellular 3D structures (Fig. 2b, cf. 2a, quantified in 2d). This was reversible as replacement of media on day 8, with media lacking IL-6 nAb, resulted in a significant increase in diameter of the MCF10.DCIS structures after an additional 48 h (Fig. 2c, cf. 2b, quantified in 2d).

To determine the effects of IL-6 nAb on MCF10.DCIS structures, we examined the expression of a panel of candidate genes that have been associated with tumor growth and invasion [39–43]. The expression of *IL-6* was upregulated 2-fold in the treated cultures. The expression of *TWIST1, vimentin*, and *Fibroblast Growth Factor 2* was downregulated greater than 2-fold, while minimal changes were observed in the expression of *E-cadherin*, *N-cadherin*, and *NFκB1* (Fig. 2e). To test whether pharmacological suppression of IL-6 could reproduce IL-6 nAb mediated growth inhibition, we treated cells with oxymatrine, a naturally occurring inhibitor of IL-6 gene expression. Oxymatrine has been shown to prevent nuclear translocation of NFκB-p65 thereby inhibiting transcriptional activation of its target genes, which include IL-6 [44]. Oxymatrine treatment was able to replicate the growth inhibitory effects observed with IL-6 nAb (Additional file 4: Figure S2B, cf. S2A, quantified in S2C). Neither oxymatrine nor IL-6 nAb treatment resulted in marked cell death as cytotoxicity assays showed no difference in cell viability after 48-hour drug treatment (Additional file 4: Figure S2D).

### Carcinoma-associated fibroblasts express IL-6 and promote DCIS cell proliferation and motility

CAFs represent a population or group of populations of stromal cells that can promote tumor cell growth [14, 45–47]. The mechanism of supported tumor growth is likely through stromal-epithelial paracrine signaling. Therefore, we next evaluated human breast CAFs to determine their contribution of IL-6 in the tumor microenvironment. Additionally, we examined the role that CAFs play in MCF10.DCIS cell proliferation and motility in the 3D MAME model.

**Fig. 3:**
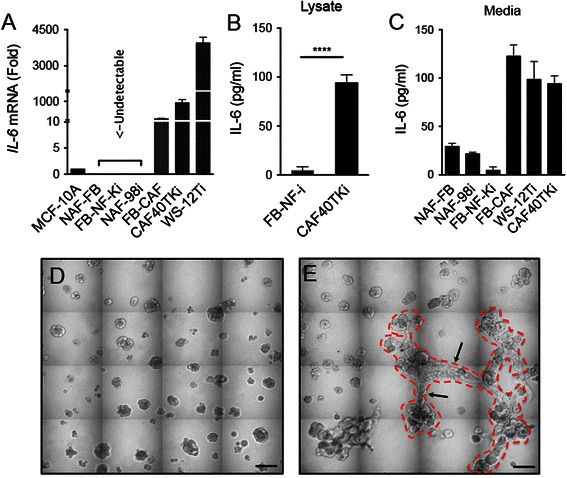
Carcinoma-associated fibroblasts (CAFs) have high expression of IL-6 and promote MCF10.DCIS growth. **a** Expression of IL-6 mRNA in three CAF cell lines (FB-CAF, CAF40TKi, WS12Ti) and three normal fibroblast cell lines (NAF-FB, FB-NF-Ki, NAF-98i) (Fold difference relative to MCF-10A non-transformed epithelial cells) (N = 3). **b-c** IL-6 protein concentration in cell lysates and media as determined by ELISA (N = 3-5) (Also see Additional file 4: Figure S2E). ****P ≤ 0.0001, Student’s *t*-test; mean ± SD **d** Representative contiguous tiled DIC image of MCF10.DCIS cells grown in MAME culture for 8 days. **e** Co-culture of MCF10.DCIS cells with CAF40TKi fibroblasts for 8 days. Dashed red line outlines multicellular structures formed in co-culture. Arrows identify interconnecting structures. Scale bars, 200 μm

We examined the expression of *IL-6* mRNA in normal human fibroblasts and CAFs grown in 3D. Here we found that CAFs exhibited elevated expression of *IL-6* mRNA compared to normal fibroblasts (Fig. 3a). Protein levels of IL-6 in FB-NF-i normal fibroblast lysates were near the lower limit of detection and undetectable in NAF-FB or NAF98i lysates. IL-6 levels in CAF40TKi lysates were significantly higher than in FB-NF-i lysates (Fig. 3b). Levels of IL-6 in CAF-conditioned media were higher than in normal fibroblast-conditioned media (Fig. 3c).

We next co-cultured MCF10.DCIS cells with CAFs in 3D, at a seeding ratio of five tumor cells to one CAF [32], to evaluate the effect of CAF-secreted cytokines on DCIS cell proliferation and related changes in morphology of multicellular structures. We found that in MCF10.DCIS:CAF40TKi co-cultures there was an increase in the average diameter and volume of the multicellular structures and a prominent formation of branch-like interconnections between the structures (Fig. 3e, cf. 3d, quantified in Additional file 5: Figure S3A). Fluorescent imaging of MCF10.DCIS:CAF40TKi 3D co-cultures revealed that the branch-like multicellular connections between structures were primarily composed of tumor cells, yet contained some CAFs (Additional file 5: Figure S3B and Additional file 6: Video S1).

We also observed that CAFs induced an increased rate of proliferation in MCF10.DCIS cells. Using a thymidine analog to evaluate the rate of DNA synthesis, we observed that co-cultures had a consistently higher rate of DNA synthesis than CAFs alone or DCIS cells alone (Additional file 7: Figure S4A-D). CAF and DCIS co-culture using a slower growing DCIS cell line, i.e., SUM102, also resulted in changes in multicellular structure formation. We observed the presence of multiple invasive processes in SUM102:CAF co-cultures that were completely absent in cultures of the SUM102 cells alone (Additional file 8: Figure S5C and D, arrow, cf. S5A and B).

### DCIS cells migrate with CAFs at the invasive front

**Fig. 4:**
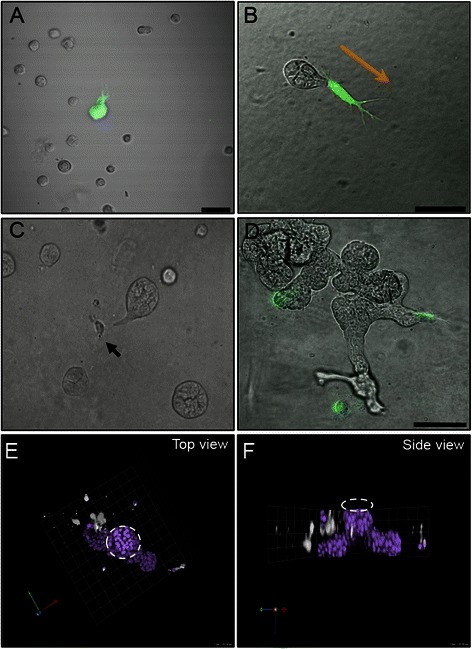
DCIS cells migrate preferentially towards CAFs in MAME co-culture. Representative DIC/fluorescent overlay image of MCF10.DCIS cells (unlabeled) with CAF40TKi fibroblasts (green) at day 1 (**a**) and 3 (**b**). Scale bar, 50 μm. Orange arrow indicates migration direction (ref. Additional file 9: Video S2). **c** Video snapshot of live cell imaging between days 3 and 4 shows a tumor spheroid attached to a single fibroblast (Arrow; ref. Additional file 9: Video S2). **d** At day 7, tumor cells have proliferated and formed protruding structures, which are connected to fibroblasts. Scale bar, 100 μm. **e** Representative 3D reconstructed image showing aerial view (**e**) and side view (**f**) of MAME MCF10.DCIS:CAF40TKi co-culture (ref. Additional file 11: Video S3). A primary MCF10.DCIS spheroid (dashed white circle) can be seen with lateral protrusions towards fibroblasts (CFSE-labeled and pseudo-colored white). Nuclei are stained with DAPI (purple). 1 Unit = 45 μm

CAFs express and secrete a number of proteases, which enhance their ability to migrate and remodel extracellular matrices [48–51]. In CAF:MCF10.DCIS co-cultures, we evaluated cell:cell interactions and motility. Here we found that MCF10.DCIS spheroids formed attachments to CAFs and remained associated with them throughout an 8-day culture. On day 1 (24 h after seeding) of a co-culture we observed many single DCIS cells and a few CSFE labeled CAFs (Fig. 4a). After 3 days in culture DCIS cells had formed small spheroids that were in contact with one or more CAFs (Fig. 4b). On day 5 we observed CAFs at the invasive edges of DCIS tumor spheroids. A representative image shows a CAF (Fig. 4c, arrow) in contact with a DCIS spheroid. A time-lapse video shows the CAF leading the DCIS spheroid (Additional file 9: Video S2). High magnification imaging shows heterocellular contact between a tumor structure and a single CAF (Additional file 10: Figure S6). We did not observe these invasive characteristics in DCIS cell grown alone, as the tumor structures tended to roll in the extracellular matrix (Additional file 11: Video S3). By day 7 CAFs were seen at the invasive edge of most tumor structures (Fig. 4d).

Using confocal microscopy we examined the 3D spatial organization of the DCIS cells in relation to CAFs. A top view of a 3D reconstruction shows an MCF10.DCIS structure (only nuclei labeled) branching out toward CFSE labeled CAFs (pseudo-colored white) (Fig. 4e, Additional file 12: Video S4). The core of this structure (dashed circle) formed shortly after seeding and grew in size before the two branching outgrowths formed. These outgrowths are reminiscent of the “strand” multicellular migration pattern previously described [52]. A side view of the same structure shows that DCIS cells migrated downward from the core structure toward the CAFs (Fig. 4f).

IL-6 protein expression in these co-cultures was detected using immunofluorescent staining (Additional file 13: Figure S7). A CFSE-labeled CAF showed high expression of IL-6, whereas the leading edge of the DCIS multicellular structure showed a gradient of IL-6 that was strongest near the attachment to the CAF (Additional file 13: Figure S7A, arrow). We also showed that MCF10.DCIS cells that do not migrate to serum-free media (Additional file 13: Figure S7D) migrated toward CAF-conditioned serum-free media (Additional file 13: Figure S7E, S7F).

### CAFs and MCF10.DCIS cells utilize cathepsin B to degrade matrix

**Fig. 5:**
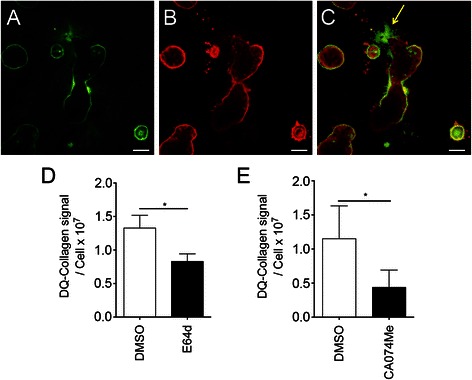
MAME co-cultures of MCF10.DCIS:CAF40TKi show expression of the cysteine protease cathepsin B at sites of proteolysis of matrix. **a** Representative confocal image obtained from the equatorial plane, showing live cell proteolysis via degraded DQ-collagen IV (dDQ-Col.IV). Fluorescent signal intensity correlates with degree of proteolysis. **b** Immunofluorescent staining for cathepsin B (red) in the equatorial plane. **c** Fluorescent overlay of dDQ-Col.IV (green) and cathepsin B immunostaining (red). Scale bar, 200 μm. Quantification of dDQ-Col.IV fluorescent intensity in MAME co-cultures treated with cell permeable inhibitors E64d (pan-cysteine protease inhibitor) (**d**) or the cathepsin B selective inhibitor CA074-Me (**e**). *P < 0.05, Student’s *t*-test; mean ± SD

IL-6 has previously been shown to upregulate cathepsin B [21], a protease associated with breast cancer progression. Therefore, we examined the expression and localization of cathepsin B in relation to active proteolysis in our 3D MAME co-cultures. Our active proteolysis assays were performed during live cell imaging [33]. Fluorescent imaging of live cells in MAME co-culture revealed regions of proteolytic activity (green) around several multicellular structures (Fig. 5a). The cells were then fixed for cathepsin B immunofluorescent staining. Visualizing the same structures revealed cathepsin B expression at the interface between the multicellular structures and the matrix (Fig. 5b). Fluorescent overlay shows co-localization between DQ-collagen IV degradation and areas with high cathepsin B expression (Fig. 5c); however, matrix degradation also occurred in areas that did not stain for cathepsin B (arrow). Upon visualization using light microscopy, the cells in these areas had morphological features consistent with CAFs, which would be consistent with current knowledge of fibroblast migration and their secretion of many proteases in addition to cathepsin B [53, 54].

We then used protease inhibitors to determine the contribution of all cysteine proteases and/or cathepsin B and L, to the overall proteolysis in the MAME culture. We treated cultures with E64d, a cell permeable pan-cysteine protease inhibitor, and found a 30 – 40 % reduction of matrix degradation (Fig. 5d). We also treated cultures with the cell permeable cathepsin B/L inhibitor, CA074Me, and found a 60 – 70 % reduction in matrix degradation (Fig. 5e). CA074Me has previously been shown to be a more efficacious inhibitor against cathepsins B and L than is E64d [35, 55]. We did not observe complete inhibition of proteolysis with the cysteine protease inhibitors, a finding in accord with previous findings that DCIS cells and CAFs secrete many non-cysteine proteases [31, 56, 57].

### Blocking IL-6 inhibits CAF-stimulated effects on human breast DCIS cells

**Fig. 6:**
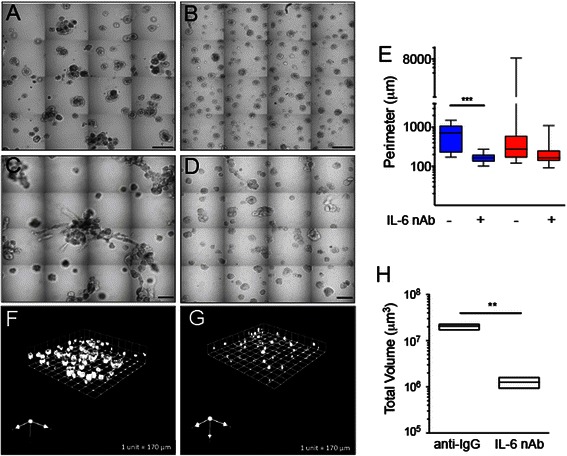
IL-6 nAb inhibits CAF-stimulated MCF10.DCIS structure growth and results in altered morphology. Contiguous 16-tile DIC image of CAFs: WS12Ti (A, B) or CAF40TKi (**c, d**) co-cultured with MCF10.DCIS cells for 8 days in the presence of isotype matched anti-IgG (**a, c**) or 1 μg /ml IL-6 nAb (**b, d**). **e** Perimeter measurements from DCIS structures in MAME culture in the presence of anti-IgG or IL-6 nAb (N = 100-140 measurements from 6 contiguous tiled DIC images (N = 3). Fluorescent tiled images of CAF40TKi fibroblasts (unlabeled) cultured with MCF10.DCIS-RFP cells in the presence of anti-IgG (**f**) or 1 μg /ml IL-6 nAb (**g**). **h** Quantification of total volume of DCIS-RFP structures in co-culture with CAF40TKi fibroblasts (ratio-paired *t*-test, N = 3). ***P ≤ 0.001, **P ≤ 0.01; Student’s *t*-test; mean ± SD

We have shown that CAFs promote the proliferation and migration of human breast DCIS cells. To determine whether CAF-stimulated migration and interaction with MCF10.DCIS cells could be inhibited by blocking IL-6, we co-cultured MCF10.DCIS cells with two human breast carcinoma-associated fibroblast lines WS-12Ti or CAF40TKi, in the presence of IL-6 nAb or an isotype control antibody. IL-6 nAb reduced the size of MCF10.DCIS:CAF multicellular structures when grown with either CAF cell line (Fig. 6a-e). Multicellular structure volume measurements confirmed a significant reduction when treated with IL-6 nAb (Fig. 6f-h).

To confirm the role of IL-6 as a key player in the formation of the multicellular structure interconnections, we utilized shRNA targeting *IL-6* expression. In CAF40TKi and MCF10.DCIS cells, we achieved greater than 50 % reduction in secreted IL-6 (Additional file 14: Figure S8A). When we co-cultured CAF40TKi-shRNA control fibroblasts with MCF10.DCIS cells, we saw a phenotype similar to non-shRNA transduced cultures (Additional file 14: Figure S8B, cf. 3E). Knocking down CAF40TKi *IL-6* expression in co-culture resulted in the formation of multicellular structures with uniform borders and few invasive processes (Additional file 14: Figure S8C). Co-culture with non-shRNA transduced CAF40TKi fibroblasts and shRNA*-IL-6* transduced MCF10.DCIS cells showed greater MCF10.DCIS:CAF40TKi interaction and multicellular structure branching (Additional file 14: Figure S8D).

IL-6 signaling is propagated through either direct cell membrane receptor signaling or soluble receptor trans-signaling (TS). In the DCIS cell lines, we found that IL-6R expression was low and sometimes undetectable by qRT-PCR. Additionally, we detected very low levels of sIL-6R secreted from MCF10.DCIS cells and none from SUM102 cells. In contrast, CAFs had a higher level of IL-6R gene expression and high secretion of sIL-6R as verified by ELISA. These findings suggest that IL-6TS is a likely mechanism of IL-6 action in these DCIS cells [58] (Additional file 15: Figure S9).

## Discussion

A number of factors produced by CAFs have been shown to be involved in promoting malignant transformation in epithelial cells, these include TGFß [59–61] and CXCL12 (SDF-1) [59, 61, 62]. These factors are involved in eliciting a range of responses that are context-dependent and that benefit the tumor in various ways. The present study describes a novel addition to these known interactions and a new mechanism by which CAF can influence tumor progression. There is a direct correlation between serum IL-6 levels and poor prognosis in breast cancer patients [36, 63–65]. Studies have shown that CAFs [66], and various immune/inflammatory cells secrete pro-inflammatory cytokines including IL-6 and contribute to tumorigenicity [67–73].

In the current study, we show that IL-6 expression can be found in both the tumor and stromal compartments. In our IHC data we found approximately 65 % of patient samples had positive IL-6 staining; however, when we examined IL-6 expression in our DCIS tumor cell lines, we found the expression to be near the lower threshold of our assay. This discrepancy may be due to differences in gene expression between tissue and cells [74], differences in IL-6 expression with tumor grade/invasiveness [65], degree of “stemness” in cell lines vs. tissue [75], and differences between the assays and/or sample collection.

We next examined the interaction between human breast DCIS epithelial cells and human breast CAFs in the context of an in vitro 3D microenvironment. We hypothesized that CAFs promoted the migration of breast DCIS cells via paracrine signaling within the tumor microenvironment. Here we observed that DCIS cells migrated towards CAFs and upon attachment to CAFs, DCIS cells remained attached and migrated through the matrix following the lead of the CAFs. Similar findings of fibroblast-led migration have been reported for co-cultures of invasive squamous carcinoma cells and CAFs [76]. It is also likely that CAFs migrated to DCIS cells, as some fibroblasts moved more easily and quickly through the matrix as seen in time-lapse videos; therefore it is possible that some fibroblasts migrated to and attached to DCIS cells thereupon leading migration of the DCIS cells.

An increase in DCIS cell proliferation and a change in multicellular structure morphology was observed in all of our co-culture experiments. DCIS multicellular structures showed invasive characteristics, having lost their uniform circular structure and had developed single or multiple protruding edges. An underlying mechanism for DCIS:CAF interaction, enhanced tumor cell proliferation, and migration was IL-6 signaling in the tumor microenvironment. We [31] as well as Sung et al. [77] have shown in 3D co-culture systems that paracrine HGF and its receptor cMet can drive the invasive potential of the MCF10.DCIS cells. We confirmed this in xenografts formed by orthotopic implantation of HGF-secreting fibroblasts and MCF10.DCIS cells in SCID mice [31]. HGF and IL-6 have been shown to cooperatively enhance lung cancer cell invasion by upregulation of their corresponding cell surface receptors [78]. Also of interest is the stem cell-like properties of the proliferating DCIS cell population as Krishnamurthy et al. have shown that endothelial IL-6 enhances self-renewal of cancer stem-like cells. Whether or not this is the case for CAF IL-6 in regard to proliferation of DCIS cells has yet to be determined [79].

Treatment of DCIS:CAF co-cultures with an IL-6 nAb abrogated the proliferation and migratory phenotype acquired by DCIS cells. This phenotype was primarily produced by the inhibition of CAF secreted IL-6 as shRNA knockdown of IL-6 in CAFs, but not in DCIS cells was able to replicate the phenotype. We also showed that MCF10.DCIS cells treated with IL-6 nAb had a down regulation of genes associated with EMT. A recent study suggests that tumor cell EMT is mediated through factors secreted from CAFs and that selective inhibition of TGFβ1 is sufficient to reverse EMT associated gene expression [80]. Other studies show cross talk between IL-6 and TGFβ signaling consistent with IL-6 and TGFβ both acting as drivers of EMT [81].

A Federal Drug Administration (FDA) approved humanized anti-IL-6R antibody, tocilizumab (Actemra), has shown promise in the treatment of inflammatory diseases particularly rheumatoid arthritis, Crohn’s disease, and Castleman’s disease. A major caveat of Actemra is that serum IL-6 levels are increased in patients after drug administration [82]. Since breast cancer patients with elevated serum IL-6 have poorer prognosis, Actemra may not be a practical therapy in these patients, however alternative approaches to reduce IL-6 signaling may prove efficacious.

Siltuximab, a monoclonal antibody against IL-6, is in clinical trials for therapies including; combinatorial treatment of metastatic renal cell carcinoma, multiple myeloma, and prostate cancer [83–86]. The FDA recently approved siltuximab for the treatment of multicentric Castleman’s disease [87]. Studies have shown that siltuximab significantly inhibits the growth of non-small-cell lung cancer in primary xenografts [88], and ovarian cancer cell xenografts [89]. Therapeutic use of siltuximab for the treatment of breast cancer has not been fully evaluated, although preliminary studies with the estrogen receptor alpha positive MCF-7 breast cancer cell line suggests potential efficacy [90].

A limitation of the current study is that we did not have complete histories or demographic data to perform correlation studies to determine how IL-6 expression in DCIS relates to tumor prognosis. Another limitation is there is a limited number of commercially available DCIS cell lines. Here we used two cell lines that are commercially available. A third DCIS cell line that is commercially available is the SUM-225 DCIS cell line; however, this line comes from a metastatic recurrence so we chose not to use it. Another limitation is that our study only followed the progression and interaction of DCIS cells with CAFs, but not inflammatory cells associated with breast tumors. Additionally, we are investigating the probability of increasing the number of cell types grown together in our 3D MAME culture, for example: macrophages, adipocytes and lymphocytes.

## Conclusion

In conclusion, we have shown that IL-6 paracrine signaling between DCIS cells and CAFs is a key mediator of early stage breast cancer cell proliferation and migration. Furthermore, IL-6 from CAFs facilitated the transition of DCIS cells from preinvasive to an invasive phenotype. This study highlights the necessity to explore paracrine signaling within the context of the tumor microenvironment and components therein.

## Additional files

Additional file 1: **Supplemental Materials and Methods.** (DOCX 126 kb)
Additional file 2: Figure S1. Schematic diagram of 3D mammary architecture and microenvironment engineering (MAME) model. In this mixed cell type model, glass bottom dishes or coverslips are coated with 100 % Cultrex and placed in a 37° 5 % CO2 incubator for 20 min. This allows the Cultrex to solidify. Here we added fibroblasts to the solidified matrix and allowed them to attach for a period of 30–45 min. After fibroblasts have adhered to the matrix tumor cells are added. An additional 30–45 min is required to allow tumor cells to adhere. After tumor cells have adhered, an overlay of 2 % Cultrex diluted with cell culture media containing 2.5 % fetal bovine serum is added. (TIFF 125 kb)
Additional file 3: Table S1. Taqman Gene Expression Assays. Taqman Assays (primers) were selected for the human gene targets. Each Taqman Assay was selected based on suppliers “best coverage” criteria. (TIFF 83 kb)
Additional file 4: Figure S2. Oxymatrine treatment. (A-B) MCF10.DCIS cells were grown in the presence of DMSO (A) or 1 mg/ml oxymatrine (B). Oxymatrine or DMSO was added 1 day after initial seeding. Scale bar, 200 μm. MCF10.DCIS cells grown in DMSO form large structures similar to untreated cultures (cf. Fig. 2a). (C) Measurement of structure diameters in three tiled images from three independent experiments. (D) Cell viability was evaluated using an ATP-based luminescence assay 24 h after drug treatment. Arbitrary units (A.U.). **P < 0.01, not significant (NS). Student’s *t*-test; mean ± SD. (TIFF 141 kb)
Additional file 5: Figure S3. Co-culture of MCF10.DCIS cells and CAFs increased MCF10.DCIS structure size and length of interconnections between multicellular structures. (A) Measurements taken from contiguous 16-tiled image of MCF10.DCIS cells alone (open box) or co-cultured (shaded box) with CAF40TKi fibroblasts showed a significant increase in the diameter of multicellular structures and length of interconnections between structures (N = 15). (B) Interconnections between multicellular structures were composed primarily of MCF10.DCIS cells. Reconstructed confocal image of MCF10.DCIS-RFP cells (red) grown in MAME co-culture with CAF40TKi-CFSE cells (green) for 8 days. CAF40TKi CFSE-labeled fibroblasts clustered near invasive edges (arrows). 20X magnification. ***P < 0.001. (TIFF 150 kb)
Additional file 6: Video S1. 360° views of a branching nodular structure consisting of MCF10.DCIS cells (red) and CAF40TKi fibroblasts (green). Image obtained on a Zeiss LSM 780 and converted to a movie file using Volocity® software. (MOV 1 mb)
Additional file 7: Figure S4. MCF10.DCIS: CAF40TKi MAME co-culture increased cell proliferation. (A) Lack of 5-Ethynyl-2´-deoxyuridine (EDU) thymidine analog incorporation in CAF40TKi fibroblasts grown for 5 days. Arrows point out fibroblasts scattered throughout the culture. Scale bar, 200 μm. (B) Incorporation of EDU in MCF10.DCIS cells grown for 5 days. (C) Incorporation of EDU in a representative structure from a MCF10.DCIS: CAF40TKi co-culture. Note that similar sized structures were chosen to compare levels of EDU incorporation in a 24-h period. Scale bars, 100 μm. (D) DNA was extracted from MAME cultures to quantify EDU concentration. Data shows a significantly higher concentration of EDU incorporation in DNA of co-culture as compared to monotypic cultures. ***P < 0.001, ****P < 0.0001, Student’s *t*-test; mean ± SD. (TIFF 344 kb)
Additional file 8: Figure S5. Carcinoma-associated fibroblasts enhance invasive growth of SUM102 cells in MAME co-culture. (A) Representative contiguous tile image of SUM102 cells grown in MAME culture for 8 days (N = 3). (B) Single field from panel A shows spheroidal SUM102 structures. (C) Representative MAME co-culture of SUM102 and CAF40TKi cells (N = 3). (D) Single field from panel C shows SUM102 cells having many invasive outgrowths (arrow). Scale bar, 50 μm (B and D), 200 μm (A and C). (TIFF 963 kb)
Additional file 9: Video S2. Video clip shows MAME co-culture of unlabeled MCF10.DCIS cells with CAF40TKi fibroblasts. Day 5 of an 8-day culture. Computer curser is used to outline a CAF (single cell) and the tumor spheroids (5 identified in video). Tumor spheroids have a characteristic rotating motion that is noticed immediately. CAFs, only two in the field of view, tend to migrate freely in the 3D matrix. The CAFs extend fingerlike cytoplasmic projections up to 100 μm in length. Time-lapse video captured using a Perkin Elmer UltraVIEW ERS confocal microscope. (MOV 28 mb)
Additional file 10: Figure S6. MCF10.DCIS multicellular structures form heterocellular contacts with CAFs. (A) An 8-day 3D MAME co-culture of MCF10.DCIS cells and CAF40TKi fibroblasts show a CFSE labeled fibroblast at the invasive edge of a multicellular DCIS structure. Scale bar equals 50 μm. (B) High magnification zoom reveals the contact region as a dense demarcation between tumor and fibroblast. Color removed to enhance visualization of heterocellular contact. (TIFF 766 kb)
Additional file 11: Video S3. Video clip of MCF10.DCIS cells on day 5 of an 8-day 3D MAME culture. The tumor cells have formed a spheroid and display a rotational motion in the extracellular matrix. Time-lapse video captured using a Perkin Elmer UltraVIEW ERS confocal microscope. (MOV 2241 kb)
Additional file 12: Video S4. Video clip shows the transition through optical slices of a MCF10.DCIS structure in co-culture with CAF40TKi fibroblasts (Full 3D image shown in Fig. 4e and f). Cultures were fixed and immunostained for IL-6 (red), with DAPI labeled nuclei. MCF10.DCIS cells formed large nodular-like structures that branched out towards CFSE-labeled CAFs (pseudo-colored white). Images were obtained on a Zeiss LSM 780 and converted to a movie file using Volocity® software. (MOV 1 mb)
Additional file 13: Figure S7. CAF-conditioned medium induces MCF10.DCIS cell migration. (A) A representative CAF40TKi fibroblast expresses IL-6 (red) as it leads an MCF10.DCIS structure during collective migration (collective migration is inferred based on observations from time-lapse microscopy, see Movie 2). An MCF10.DCIS structure (arrow) shows a gradient of IL-6 expression (red) that is most intense near a cell:cell attachment with a CAF40TKi fibroblast. (B) DIC/fluorescent overlay image identifies the single leading cell as a CFSE-labeled CAF40TKi fibroblast (green). MCF10.DCIS cells are unlabeled. (C) Fluorescence only overlay of panels A and B. Scale bar, 20 μm. (D) A migration assay membrane showed that MCF10.DCIS cells do not migrate through transwell membrane pores to serum-free media. (E) MCF10.DCIS cell migrate through the transwell membrane pores to CAF40TKi conditioned serum-free media. (F) Quantification of visible cells that have migrated through transwell membrane filter after 24 h. (TIFF 11394 kb)
Additional file 14: Figure S8. Knockdown of IL-6 in CAF40TKi fibroblasts inhibits their interaction with MCF10.DCIS cells. (A) CAF40TKi fibroblasts and MCF10.DCIS cells were transduced with an shRNA targeting IL-6 mRNA. ELISA quantification of IL-6 secreted from control (black bars) and IL-6 targeted shRNA cell lines (red bars). (B) MCF10.DCIS cells co-cultured with CAF40TKi fibroblasts transduced with a scrambled shRNA virus. (C) Co-culture of MCF10.DCIS cells (unlabeled) with IL-6 shRNA transduced CAF40TKi fibroblasts (green). (D) Co-culture of IL-6 shRNA transduced MCF10.DCIS cells (red) with CAF40TKi fibroblasts (unlabeled). Scale bars, 200 μm. ***P < 0.001. All statistics are Student’s *t*-test; mean ± SD. (TIFF 319 kb)
Additional file 15: Figure S9. IL-6 receptor gene expression and protein secretion in DCIS cell lines and CAF40TKi fibroblasts. (A) Examination of IL-6R gene expression. Fold difference as compared to MCF-10A non-tumor-forming breast epithelial cells. (B) Measurement of soluble IL-6R in media collected from MAME cultures (determined by ELISA). **P ≤ 0.001, NS. Not significant, by Student’s *t*-test; mean ± SD. (TIFF 119 kb)

## Abbreviations

DCIS: Ductal carcinoma in situ
CAFs: Carcinoma-associated fibroblasts
IL-6: Interleukin 6
IL-6TS: Interleukin 6– trans-signaling
MAME: Mammary architecture and microenvironment engineering
CFSE: Carboxyfluorescein diacetate succinimidyl ester
dDQ-col: Degraded dye-quenched collagen
3D: Three-dimensional
EMT: Epithelial to mesenchymal transition
ELISA: Enzyme-linked immunosorbent assay
qRT-PCR: Quantative Real-time polymerase chain reaction
shRNA: Short hairpin ribonucleic acid
LSM: Laser scanning microscope
DIC: Differential interference contrast
Oxy: Oxymatrine
DMSO: Dimethyl sulfoxide
DNA: Deoxyribonucleic acid
FDA: Federal Drug Administration

## References

[1] Virnig BA, Tuttle TM, Shamliyan T, Kane RL (2010). Ductal carcinoma in situ of the breast: a systematic review of incidence, treatment, and outcomes. J Natl Cancer Inst.

[2] Society AC. Cancer Facts & Figures 2014. American Cancer Society*.* Cancer.org. 2014;8–10.

[3] Sanders ME, Schuyler PA, Dupont WD, Page DL (2005). The natural history of low-grade ductal carcinoma in situ of the breast in women treated by biopsy only revealed over 30 years of long-term follow-up. Cancer.

[4] Page DL, Dupont WD, Rogers LW, Jensen RA, Schuyler PA (1995). Continued local recurrence of carcinoma 15–25 years after a diagnosis of low grade ductal carcinoma in situ of the breast treated only by biopsy. Cancer.

[5] Network NCC (2014). Stage 0 Breast Cancer. NCCN Guidlines for Patients.

[6] Bissell MJ, Radisky DC, Rizki A, Weaver VM, Petersen OW (2002). The organizing principle: microenvironmental influences in the normal and malignant breast. Differentiation.

[7] Lee GY, Kenny PA, Lee EH, Bissell MJ (2007). Three-dimensional culture models of normal and malignant breast epithelial cells. Nat Methods.

[8] Ma XJ, Dahiya S, Richardson E, Erlander M, Sgroi DC (2009). Gene expression profiling of the tumor microenvironment during breast cancer progression. Breast Cancer Res.

[9] Hu M, Yao J, Cai L, Bachman KE, van den Brule F, Velculescu V, et al. Distinct epigenetic changes in the stromal cells of breast cancers. Nat Genet. 2005;37(8):899–905. 10.1038/ng1596 16007089

[10] Sung KE, Yang N, Pehlke C, Keely PJ, Eliceiri KW, Friedl A, et al. Transition to invasion in breast cancer: a microfluidic in vitro model enables examination of spatial and temporal effects. Integr Biol. 2011;3(4):439–50. 10.1039/c0ib00063a PMC3094750 21135965

[11] Madar S, Goldstein I, Rotter V (2013). Cancer associated fibroblasts’--more than meets the eye. Trends Mol Med.

[12] Campbell I, Polyak K, Haviv I (2009). Clonal mutations in the cancer-associated fibroblasts: the case against genetic coevolution. Cancer Res.

[13] Qiu W, Hu M, Sridhar A, Opeskin K, Fox S, Shipitsin M, et al. No evidence of clonal somatic genetic alterations in cancer-associated fibroblasts from human breast and ovarian carcinomas. Nat Genet. 2008;40(5):650–5. 10.1038/ng.117 PMC3745022 18408720

[14] Augsten M (2014). Cancer-associated fibroblasts as another polarized cell type of the tumor microenvironment. Front Oncol..

[15] Tamm I, Cardinale I, Murphy JS (1991). Decreased adherence of interleukin 6-treated breast carcinoma cells can lead to separation from neighbors after mitosis. Proc Natl Acad Sci U S A.

[16] Tamm I, Cardinale I, Kikuchi T, Krueger JG (1994). E-cadherin distribution in interleukin 6-induced cell-cell separation of ductal breast carcinoma cells. Proc Natl Acad Sci U S A.

[17] Krueger J, Ray A, Tamm I, Sehgal PB (1991). Expression and function of interleukin-6 in epithelial cells. J Cell Biochem.

[18] Cheng GZ, Zhang WZ, Sun M, Wang Q, Coppola D, Mansour M, et al. Twist is transcriptionally induced by activation of STAT3 and mediates STAT3 oncogenic function. J Biol Chem. 2008;283(21):14665–73. 10.1074/jbc.M707429200 PMC2386910 18353781

[19] Sullivan NJ, Sasser AK, Axel AE, Vesuna F, Raman V, Ramirez N, et al. Interleukin-6 induces an epithelial-mesenchymal transition phenotype in human breast cancer cells. Oncogene. 2009;28(33):2940–7. 10.1038/onc.2009.180 PMC5576031 19581928

[20] Rose-John S, Heinrich PC (1994). Soluble receptors for cytokines and growth factors: generation and biological function. Biochem J.

[21] Mohamed MM, Cavallo-Medved D, Rudy D, Anbalagan A, Moin K, Sloane BF (2010). Interleukin-6 increases expression and secretion of cathepsin B by breast tumor-associated monocytes. Cell Physiol Biochem.

[22] Bengsch F, Buck A, Gunther SC, Seiz JR, Tacke M, Pfeifer D, et al. Cell type-dependent pathogenic functions of overexpressed human cathepsin B in murine breast cancer progression. Oncogene. 2013. 10.1038/onc.2013.395 PMC4139469 24077280

[23] Hobisch A, Rogatsch H, Hittmair A, Fuchs D, Bartsch Jr G, Klocker H, et al. Immunohistochemical localization of interleukin-6 and its receptor in benign, premalignant and malignant prostate tissue. J Pathol. 2000;191(3):239–44. 10.1002/1096-9896(2000)9999:9999<::AID-PATH633>3.0.CO;2-X 10878544

[24] Kinoshita T, Ito H, Miki C (1999). Serum interleukin-6 level reflects the tumor proliferative activity in patients with colorectal carcinoma. Cancer.

[25] Guo Y, Xu F, Lu T, Duan Z, Zhang Z (2012). Interleukin-6 signaling pathway in targeted therapy for cancer. Cancer Treat Rev.

[26] Hartman ZC, Poage GM, den Hollander P, Tsimelzon A, Hill J, Panupinthu N, et al. Growth of triple-negative breast cancer cells relies upon coordinate autocrine expression of the proinflammatory cytokines IL-6 and IL-8. Cancer Res. 2013;73(11):3470–80. 10.1158/0008-5472.CAN-12-4524-T PMC3853111 23633491

[27] Jiang XP, Yang DC, Elliott RL, Head JF (2011). Down-regulation of expression of interleukin-6 and its receptor results in growth inhibition of MCF-7 breast cancer cells. Anticancer Res.

[28] Leslie K, Gao SP, Berishaj M, Podsypanina K, Ho H, Ivashkiv L, et al. Differential interleukin-6/Stat3 signaling as a function of cellular context mediates Ras-induced transformation. Breast Cancer Res. 2010;12(5):R80. 10.1186/bcr2725 PMC3096973 20929542

[29] Lovitt CJ, Shelper TB, Avery VM (2015). Evaluation of chemotherapeutics in a three-dimensional breast cancer model. J Cancer Res Clin Oncol.

[30] Dawson PJ, Wolman SR, Tait L, Heppner GH, Miller FR (1996). MCF10AT: a model for the evolution of cancer from proliferative breast disease. Am J Pathol.

[31] Jedeszko C, Victor BC, Podgorski I, Sloane BF (2009). Fibroblast hepatocyte growth factor promotes invasion of human mammary ductal carcinoma in situ. Cancer Res.

[32] Sameni M, Anbalagan A, Olive MB, Moin K, Mattingly RR, Sloane BF. MAME models for 4D live-cell imaging of tumor: microenvironment interactions that impact malignant progression. J Vis Exp. 2012;(60). doi: 10.3791/3661. 10.3791/3661 PMC3376933 22371028

[33] Cavallo-Medved D, Rudy D, Blum G, Bogyo M, Caglic D, Sloane BF (2009). Live-cell imaging demonstrates extracellular matrix degradation in association with active cathepsin B in caveolae of endothelial cells during tube formation. Exp Cell Res.

[34] Sloane BF, Moin K, Sameni M, Tait LR, Rozhin J, Ziegler G (1994). Membrane association of cathepsin B can be induced by transfection of human breast epithelial cells with c-Ha-ras oncogene. J Cell Sci.

[35] Mullins SR, Sameni M, Blum G, Bogyo M, Sloane BF, Moin K (2012). Three-dimensional cultures modeling premalignant progression of human breast epithelial cells: role of cysteine cathepsins. Biol Chem.

[36] Dethlefsen C, Hojfeldt G, Hojman P (2013). The role of intratumoral and systemic IL-6 in breast cancer. Breast Cancer Res Treat.

[37] Bachelot T, Ray-Coquard I, Menetrier-Caux C, Rastkha M, Duc A, Blay JY (2003). Prognostic value of serum levels of interleukin 6 and of serum and plasma levels of vascular endothelial growth factor in hormone-refractory metastatic breast cancer patients. Br J Cancer.

[38] Salgado R, Junius S, Benoy I, Van Dam P, Vermeulen P, Van Marck E, et al. Circulating interleukin-6 predicts survival in patients with metastatic breast cancer. Int J Cancer. 2003;103(5):642–6. 10.1002/ijc.10833 12494472

[39] Yang L, Han S, Sun Y (2014). An IL6-STAT3 loop mediates resistance to PI3K inhibitors by inducing epithelial-mesenchymal transition and cancer stem cell expansion in human breast cancer cells. Biochem Biophys Res Commun.

[40] Hung SY, Shih YP, Chen M, Lo SH (2014). Up-regulated cten by FGF2 contributes to FGF2-mediated cell migration. Mol Carcinog.

[41] Patel NA, Patel PS, Vora HH. Role of PRL-3, Snail, Cytokeratin and Vimentin expression in epithelial mesenchymal transition in breast carcinoma. Breast Dis. 2014;35(2):113–127. 10.3233/BD-140395 25547164

[42] D’Angelo RC, Liu XW, Najy AJ, Jung YS, Won J, Chai KX, et al. TIMP-1 via TWIST1 induces EMT phenotypes in human breast epithelial cells. Mol Cancer Res. 2014;12(9):1324–33. 10.1158/1541-7786.MCR-14-0105 PMC4171133 24895412

[43] Ibrahim SA, Hassan H, Vilardo L, Kumar SK, Kumar AV, Kelsch R, et al. Syndecan-1 (CD138) modulates triple-negative breast cancer stem cell properties via regulation of LRP-6 and IL-6-mediated STAT3 signaling. PLoS One. 2013;8(12), e85737. 10.1371/journal.pone.0085737 PMC3877388 24392029

[44] Guzman JR, Koo JS, Goldsmith JR, Muhlbauer M, Narula A, Jobin C (2013). Oxymatrine prevents NF-kappaB nuclear translocation and ameliorates acute intestinal inflammation. Sci Rep.

[45] Cirri P, Chiarugi P (2011). Cancer associated fibroblasts: the dark side of the coin. Am J Cancer Res.

[46] Orimo A, Weinberg RA (2006). Stromal fibroblasts in cancer: a novel tumor-promoting cell type. Cell Cycle.

[47] Olumi AF, Grossfeld GD, Hayward SW, Carroll PR, Tlsty TD, Cunha GR (1999). Carcinoma-associated fibroblasts direct tumor progression of initiated human prostatic epithelium. Cancer Res.

[48] Chang HY, Chi JT, Dudoit S, Bondre C, van de Rijn M, Botstein D, et al. Diversity, topographic differentiation, and positional memory in human fibroblasts. Proc Natl Acad Sci U S A. 2002;99(20):12877–82. 10.1073/pnas.162488599 PMC130553 12297622

[49] Rowe RG, Keena D, Sabeh F, Willis AL, Weiss SJ (2011). Pulmonary fibroblasts mobilize the membrane-tethered matrix metalloprotease, MT1-MMP, to destructively remodel and invade interstitial type I collagen barriers. Am J Physiol Lung Cell Mol Physiol.

[50] Holliday DL, Hughes S, Shaw JA, Walker RA, Jones JL (2007). Intrinsic genetic characteristics determine tumor-modifying capacity of fibroblasts: matrix metalloproteinase-3 5A/5A genotype enhances breast cancer cell invasion. Breast Cancer Res.

[51] Casey T, Bond J, Tighe S, Hunter T, Lintault L, Patel O, et al. Molecular signatures suggest a major role for stromal cells in development of invasive breast cancer. Breast Cancer Res Treat. 2009;114(1):47–62. 10.1007/s10549-008-9982-8 18373191

[52] Friedl P, Alexander S (2011). Cancer invasion and the microenvironment: plasticity and reciprocity. Cell.

[53] Egeblad M, Werb Z (2002). New functions for the matrix metalloproteinases in cancer progression. Nat Rev Cancer.

[54] O’Brien P, O’Connor BF (2008). Seprase: an overview of an important matrix serine protease. Biochim Biophys Acta.

[55] Linebaugh BE, Sameni M, Day NA, Sloane BF, Keppler D (1999). Exocytosis of active cathepsin B enzyme activity at pH 7.0, inhibition and molecular mass. Eur J Biochem.

[56] Nielsen BS, Rank F, Lopez JM, Balbin M, Vizoso F, Lund LR, et al. Collagenase-3 expression in breast myofibroblasts as a molecular marker of transition of ductal carcinoma in situ lesions to invasive ductal carcinomas. Cancer Res. 2001;61(19):7091–100. 11585740

[57] Kessenbrock K, Plaks V, Werb Z (2010). Matrix metalloproteinases: regulators of the tumor microenvironment. Cell.

[58] Peters M, Muller AM, Rose-John S (1998). Interleukin-6 and soluble interleukin-6 receptor: direct stimulation of gp130 and hematopoiesis. Blood.

[59] Ao M, Franco OE, Park D, Raman D, Williams K, Hayward SW (2007). Cross-talk between paracrine-acting cytokine and chemokine pathways promotes malignancy in benign human prostatic epithelium. Cancer Res.

[60] Franco OE, Jiang M, Strand DW, Peacock J, Fernandez S, Jackson 2nd RS, et al. Altered TGF-beta signaling in a subpopulation of human stromal cells promotes prostatic carcinogenesis. Cancer Res. 2011;71(4):1272–81. 10.1158/0008-5472.CAN-10-3142 PMC3076790 21303979

[61] Hendrayani SF, Al-Khalaf HH, Aboussekhra A. The Cytokine IL-6 Reactivates Breast Stromal Fibroblasts through Transcription Factor STAT3-dependent Up-regulation of the RNA Binding Protein AUF1. J Biol Chem. 2014. 10.1074/jbc.M114.594044 PMC4223303 25231991

[62] Orimo A, Gupta PB, Sgroi DC, Arenzana-Seisdedos F, Delaunay T, Naeem R, et al. Stromal fibroblasts present in invasive human breast carcinomas promote tumor growth and angiogenesis through elevated SDF-1/CXCL12 secretion. Cell. 2005;121(3):335–48. 10.1016/j.cell.2005.02.034 15882617

[63] Cho YA, Sung MK, Yeon JY, Ro J, Kim J (2013). Prognostic role of interleukin-6, interleukin-8, and leptin levels according to breast cancer subtype. Cancer Res Treat.

[64] Narita D, Seclaman E, Ursoniu S, Ilina R, Cireap N, Anghel A (2011). Expression of CCL18 and interleukin-6 in the plasma of breast cancer patients as compared with benign tumor patients and healthy controls. Rom J Morphol Embryol..

[65] Ravishankaran P, Karunanithi R (2011). Clinical significance of preoperative serum interleukin-6 and C-reactive protein level in breast cancer patients. World J Surg Oncol.

[66] Franco OE, Shaw AK, Strand DW, Hayward SW (2010). Cancer associated fibroblasts in cancer pathogenesis. Semin Cell Dev Biol.

[67] Laoui D, Movahedi K, Van Overmeire E, Van den Bossche J, Schouppe E, Mommer C, et al. Tumor-associated macrophages in breast cancer: distinct subsets, distinct functions. Int J Dev Biol. 2011;55(7–9):861–7. 10.1387/ijdb.113371dl 22161841

[68] Medrek C, Ponten F, Jirstrom K, Leandersson K (2012). The presence of tumor associated macrophages in tumor stroma as a prognostic marker for breast cancer patients. BMC Cancer.

[69] Rudnick JA, Kuperwasser C (2012). Stromal biomarkers in breast cancer development and progression. Clin Exp Metastasis.

[70] Palucka K, Coussens LM, O’Shaughnessy J (2013). Dendritic cells, inflammation, and breast cancer. Cancer J.

[71] Kees T, Egeblad M (2011). Innate immune cells in breast cancer--from villains to heroes?. J Mammary Gland Biol Neoplasia.

[72] Fridman WH, Galon J, Pages F, Tartour E, Sautes-Fridman C, Kroemer G (2011). Prognostic and predictive impact of intra- and peritumoral immune infiltrates. Cancer Res.

[73] DeNardo DG, Coussens LM (2007). Inflammation and breast cancer. Balancing immune response: crosstalk between adaptive and innate immune cells during breast cancer progression. Breast Cancer Res.

[74] Wilson KS, Roberts H, Leek R, Harris AL, Geradts J (2002). Differential gene expression patterns in HER2/neu-positive and -negative breast cancer cell lines and tissues. Am J Pathol.

[75] Kim G, Ouzounova M, Quraishi AA, Davis A, Tawakkol N, Clouthier SG, et al. SOCS3-mediated regulation of inflammatory cytokines in PTEN and p53 inactivated triple negative breast cancer model. Oncogene. 2015;34(6):671–80. 10.1038/onc.2014.4 PMC4285772 24531711

[76] Gaggioli C, Hooper S, Hidalgo-Carcedo C, Grosse R, Marshall JF, Harrington K, et al. Fibroblast-led collective invasion of carcinoma cells with differing roles for RhoGTPases in leading and following cells. Nat Cell Biol. 2007;9(12):1392–400. 10.1038/ncb1658 18037882

[77] Sung KE, Su X, Berthier E, Pehlke C, Friedl A, Beebe DJ (2013). Understanding the impact of 2D and 3D fibroblast cultures on in vitro breast cancer models. PLoS One.

[78] To Y, Dohi M, Matsumoto K, Tanaka R, Sato A, Nakagome K, et al. A two-way interaction between hepatocyte growth factor and interleukin-6 in tissue invasion of lung cancer cell line. Am J Respir Cell Mol Biol. 2002;27(2):220–6. 10.1165/ajrcmb.27.2.4804 12151314

[79] Krishnamurthy S, Warner KA, Dong Z, Imai A, Nor C, Ward BB, et al. Endothelial interleukin-6 defines the tumorigenic potential of primary human cancer stem cells. Stem Cells. 2014;32(11):2845–57. 10.1002/stem.1793 PMC4198458 25078284

[80] Yu Y, Xiao CH, Tan LD, Wang QS, Li XQ, Feng YM (2014). Cancer-associated fibroblasts induce epithelial-mesenchymal transition of breast cancer cells through paracrine TGF-beta signalling. Br J Cancer.

[81] Zhang XL, Topley N, Ito T, Phillips A (2005). Interleukin-6 regulation of transforming growth factor (TGF)-beta receptor compartmentalization and turnover enhances TGF-beta1 signaling. J Biol Chem.

[82] Nishimoto N, Terao K, Mima T, Nakahara H, Takagi N, Kakehi T (2008). Mechanisms and pathologic significances in increase in serum interleukin-6 (IL-6) and soluble IL-6 receptor after administration of an anti-IL-6 receptor antibody, tocilizumab, in patients with rheumatoid arthritis and Castleman disease. Blood.

[83] Voorhees PM, Manges RF, Sonneveld P, Jagannath S, Somlo G, Krishnan A, et al. A phase 2 multicentre study of siltuximab, an anti-interleukin-6 monoclonal antibody, in patients with relapsed or refractory multiple myeloma. Br J Haematol. 2013;161(3):357–66. 10.1111/bjh.12266 PMC5837861 23432640

[84] Rossi JF, Negrier S, James ND, Kocak I, Hawkins R, Davis H, et al. A phase I/II study of siltuximab (CNTO 328), an anti-interleukin-6 monoclonal antibody, in metastatic renal cell cancer. Br J Cancer. 2010;103(8):1154–62. 10.1038/sj.bjc.6605872 PMC2967052 20808314

[85] Hunsucker SA, Magarotto V, Kuhn DJ, Kornblau SM, Wang M, Weber DM, et al. Blockade of interleukin-6 signalling with siltuximab enhances melphalan cytotoxicity in preclinical models of multiple myeloma. Br J Haematol. 2011;152(5):579–92. 10.1111/j.1365-2141.2010.08533.x PMC3402914 21241278

[86] Dorff TB, Goldman B, Pinski JK, Mack PC, Lara Jr PN, Van Veldhuizen Jr PJ, et al. Clinical and correlative results of SWOG S0354: a phase II trial of CNTO328 (siltuximab), a monoclonal antibody against interleukin-6, in chemotherapy-pretreated patients with castration-resistant prostate cancer. Clin Cancer Re. 2010;16(11):3028–34. 10.1158/1078-0432.CCR-09-3122 PMC2898710 20484019

[87] Johnson and Johnson (Janssen Biotech I. SYLVANT™ (siltuximab) Receives FDA Approval to Treat Multicentric Castleman’s Disease (MCD). www.jnj.com/news/all/sylvant. 2014.

[88] Song L, Smith MA, Doshi P, Sasser K, Fulp W, Altiok S, et al. Antitumor efficacy of the anti-interleukin-6 (IL-6) antibody siltuximab in mouse xenograft models of lung cancer. J Thorac Oncol. 2014;9(7):974–82. 10.1097/JTO.0000000000000193 PMC4057975 24922005

[89] Coward J, Kulbe H, Chakravarty P, Leader D, Vassileva V, Leinster DA, et al. Interleukin-6 as a therapeutic target in human ovarian cancer. Clin Cancer Res. 2011;17(18):6083–96. 10.1158/1078-0432.CCR-11-0945 PMC3182554 21795409

[90] Axel A, Casneuf T, King P, Alvarez J, Hall B, Sasser K. Abstract 3530: The role of IL-6 in ER + breast cancer and potential use for Siltuximab, an anti-IL-6 antibody, in ER + breast cancer treatment. Cancer Res. 2013;73(8 Supplement):3530–0.

