# Supplementary material for: Il-6 signaling between ductal carcinoma in situ cells and carcinoma-associated fibroblasts mediates tumor cell growth and migration
Source: BMC Cancer. 2015 Aug 13;15:584. doi: 10.1186/s12885-015-1576-3 (PMC4535667; doi:10.1186/s12885-015-1576-3)
Supplement: Additional file 1: — Supplemental Materials and Methods. (DOCX 126 kb) [file 12885_2015_1576_MOESM1_ESM.docx]

**Supplemental Materials and Methods**

**Cell Lines and Cell Culture.** Human normal fibroblasts (FB-NF-i, NAF-98i, NAF-FB) were maintained in the following media: DMEM, 20% fetal bovine serum (FBS), 1% pen/strep, and 2.5 mM L-glutamine (Life Technologies). Carcinoma-associated fibroblasts (CAF40TKi, FB-CAF) were maintained in MCDB 131 without glutamine, 10% FBS v/v, 1% MEM non-essential amino acids solution 100X, 1% insulin/transferrin/selenium/sodium pyruvate solution also known as ITS-A v/v, 12% AminoMax C-100 Basal Medium v/v, 5% AminoMax C-100 Supplement v/v, 1% pen/strep, and 2.5 mM L-glutamine (Life Technologies); and WS-12Ti fibroblasts in DMEM F12, 10% FBS (Invitrogen), 1% pen/strep, and 2.5 mM L-glutamine (Sigma).

**Generation of immortalized fibroblasts.** Normal fibroblasts (FB-NF-i and NAF-98i) and CAFs (CAF40TKi) were transduced using a lentivirus that expresses hTERT and a puromycin selection marker. Briefly, cells were grown to 70% confluence, then washed 3 times with PBS before the addition of 1:1 ratio of hTERT viral supernatant (ABMGood, G200) to complete media containing 10 μg/ml polybrene (TR-1003-G, Millipore). The cells were maintained in this mixture for 36 hours then washed 3 times and returned to complete media for 48 hours. Selection of hTERT expressing cells was performed one week after transduction, using 8 μg/ ml puromycin (Life Technologies) in culture media for 3 days.

**Gene Expression.** For 3D cultures, cells were cultured in MAME culture model in either 40 mm glass bottom or 60 mm polystyrene dishes. The cultures were then washed 3 times with sterile PBS and then transferred to 15-ml conical tubes, washed in cold PBS-EDTA 3 times, 20 minutes each on ice and then pelleted for resuspension in TRIzol®. After extraction, 1 μg RNA was DNAse treated (M6101, Promega) prior to cDNA synthesis. All qRT-PCR reactions were performed using Taqman Assays (Life Technologies).

**Immunohistochemistry.** Tissue slides were deparaffinized then incubated in 3% hydrogen peroxide to block endogenous peroxidase activity. Antigens were retrieved in a microwave oven with a capacity of 650-720 W in a 10 mM citrate buffer at pH 6 for 5 minutes and then cooled for 20 minutes at room temperature. Slides were washed in PBS and then incubated for 1 hour in 2.5% goat preimmune serum (Life Technologies). IL-6 primary antibody (AF-206-NA, R&D Systems) was added at a concentration of 0.5 μg/ml for overnight incubation at 4°C. The slides were washed followed by addition of biotinylated secondary (1:1000) or fluorescent conjugated antibody (1:10,000) for 1 hour at room temperature. Post secondary washing was followed by peroxidase substrate (Vector NovaRed) for 5-8 minutes. Slides were then washed, counterstained with hematoxylin and mounted for microscopy.

**Immunofluorescence.** Nuclei were labeled with Hoechst (33342, Thermo Scientific) and EDU (Life Technologies). Polyclonal antibodies to human IL-6 (AF-206-NA, R&D Systems) were used at a concentration of 1 μg/ml. Mono-specific antibodies to human cathepsin B were previously isolated and characterized [[81](#_ENREF_81)]. Cathepsin B immunostaining was performed as previously described with the exception that 0.01% saponin was replaced with 1% Tween 20 [[82](#_ENREF_82)]. CAF40TKi were pre-labeled prior to seeding in some MAME co-cultures, utilizing CellTrace CFSE (carboxyfluorescein diacetate succinimidyl ester; Life Technologies) according to manufacturers protocol.

**Drug treatments.** For treatment of MAME cultures with IL-6 nAb (R&D, AF-206-NA), we added 1 μg/ml of IL-6 nAb in the 2% Cultrex overlay to 3D cultures on the first day of culture and refreshed with IL-6 nAb and 2% overlay every 4 days. Antibody concentration was selected based on preliminary studies determining the lowest concentration needed to significantly inhibit tumor cell proliferation. Oxymatrine (Sigma) 1-mg/ml (3.7 mM) was added 24 hours after cell seeding and was replaced with fresh drug every 4 days. Oxymatrine concentration was determined empirically based on the concentration at which proliferation was inhibited to 50% of control. The protease inhibitors CA074Me and E64d (Sigma) were used at a concentration of 10 μM.

**Migration Assay.** Migration assays were performed in under serum-free conditions on 24-well plates using BD-BioCoat^TM^ 8.0 µm pore Transwell migration filters. Lower chambers of control and test wells contained either serum-free media or 24-hour CAF40TKi-conditioned serum-free media, respectively. DCIS cells (5 x 10^3^) were cultured in upper wells for 24 hours at 37 °C and 5% CO_2_. Transwell filters were washed, Geimsa stained and imaged by light microscopy. Cells were counted in three fields of view at 10X magnification.

**Toxicity Assay.** To determine drug toxicity we used CellTiter-Glo® 3D Viability Assay (Promega). Briefly, cells were cultured using the MAME methodology in 96-well plates in the presence of either IL-6 nAb, oxymatrine, or appropriate controls (anti-IgG or DMSO) for a period of 48 hours. Cell viability was determined via a luciferase-based reaction, which generates a luminescent signal in the presence of viable cells. Luminescence was captured with a FujiFilm LAS-4000 and images analyzed using ImageJ software.

**Proliferation Assays.** Cell proliferation in MAME cultures was assessed using the Click-it EDU system (Life Technologies). DNA was extracted from cultures following fluorescent imaging for measurement of total DNA and EDU (5-ethynyl-2´-deoxyuridine) concentrations via optical absorbance.

**Knockdown of IL-6.** Translation of *IL-6* in CAF40TKi and MCF10.DCIS cells was inhibited using an IL-6 shRNA lentiviral construct obtained from Open Biosystems (Thermo Scientific). The cells were transduced using the following clones; ID: V3LHS-390095, V2LHS-111643, V3LHS-390097, and control scrambled shRNA. Only clone V3LHS-390095 was used for knockdown experiments as it produced the most effective down-regulation of IL-6. Cells were grown under standard conditions (listed in Cell Lines and Cell Culture Methods) in a 6-well plate until 60% confluence. Growth media were replaced with 1 ml growth media containing 8 μg/ml polybrene (Millipore) plus 1 ml *IL-6* shRNA lentiviral particles in FBS. Cells were incubated in a 37°C and 5% CO_2_ incubator for 2 days, then washed with PBS and returned to growth media. Knockdown was confirmed by qRT-PCR and ELISA.
