# Supplementary figures and images for: Il-6 signaling between ductal carcinoma in situ cells and carcinoma-associated fibroblasts mediates tumor cell growth and migration
Source: BMC Cancer. 2015 Aug 13;15:584. doi: 10.1186/s12885-015-1576-3 (PMC4535667; doi:10.1186/s12885-015-1576-3)

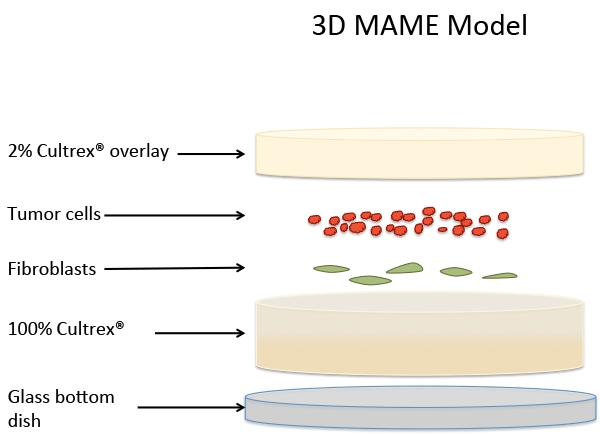

Supplement: Additional file 2: Figure S1. — Schematic diagram of 3D mammary architecture and microenvironment engineering (MAME) model. In this mixed cell type model, glass bottom dishes or coverslips are coated with 100 % Cultrex and placed in a 37° 5 % CO2 incubator for 20 min. This allows the Cultrex to solidify. Here we added fibroblasts to the solidified matrix and allowed them to attach for a period of 30–45 min. After fibroblasts have adhered to the matrix tumor cells are added. An additional 30–45 min is required to allow tumor cells to adhere. After tumor cells have adhered, an overlay of 2 % Cultrex diluted with cell culture media containing 2.5 % fetal bovine serum is added. (TIFF 125 kb) [file 12885_2015_1576_MOESM2_ESM.tiff]

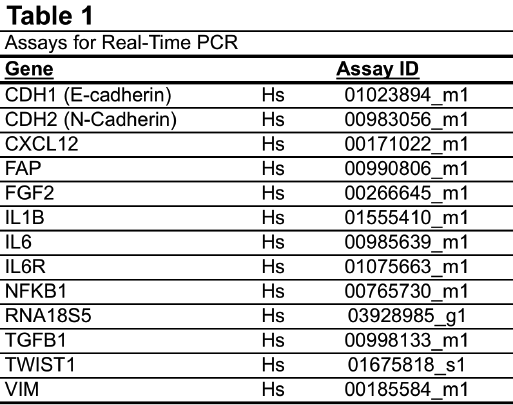

Supplement: Additional file 3: Table S1. — Taqman Gene Expression Assays. Taqman Assays (primers) were selected for the human gene targets. Each Taqman Assay was selected based on suppliers “best coverage” criteria. (TIFF 83 kb) [file 12885_2015_1576_MOESM3_ESM.tiff]

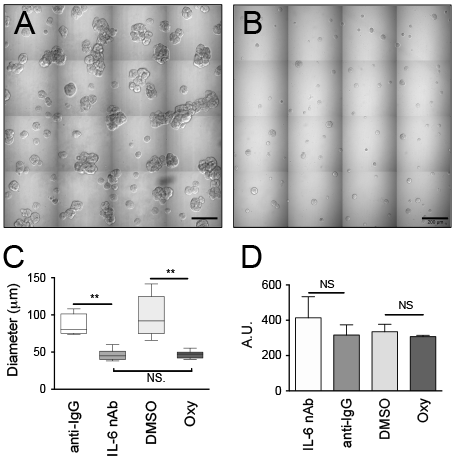

Supplement: Additional file 4: Figure S2. — Oxymatrine treatment. (A-B) MCF10.DCIS cells were grown in the presence of DMSO (A) or 1 mg/ml oxymatrine (B). Oxymatrine or DMSO was added 1 day after initial seeding. Scale bar, 200 μm. MCF10.DCIS cells grown in DMSO form large structures similar to untreated cultures (cf. Fig. 2a). (C) Measurement of structure diameters in three tiled images from three independent experiments. (D) Cell viability was evaluated using an ATP-based luminescence assay 24 h after drug treatment. Arbitrary units (A.U.). **P < 0.01, not significant (NS). Student’s t-test; mean ± SD. (TIFF 141 kb) [file 12885_2015_1576_MOESM4_ESM.tiff]

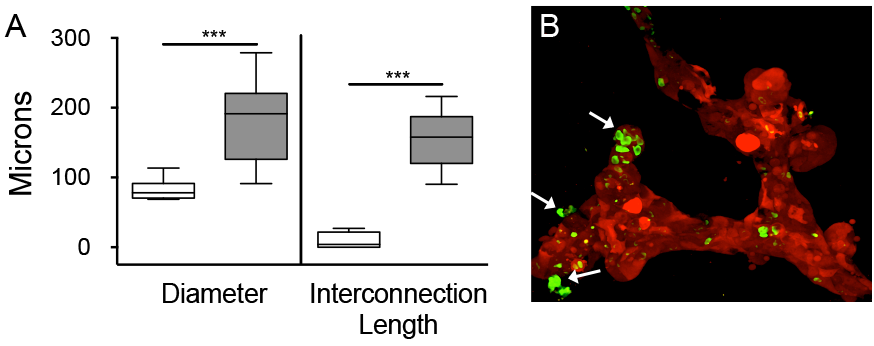

Supplement: Additional file 5: Figure S3. — Co-culture of MCF10.DCIS cells and CAFs increased MCF10.DCIS structure size and length of interconnections between multicellular structures. (A) Measurements taken from contiguous 16-tiled image of MCF10.DCIS cells alone (open box) or co-cultured (shaded box) with CAF40TKi fibroblasts showed a significant increase in the diameter of multicellular structures and length of interconnections between structures (N = 15). (B) Interconnections between multicellular structures were composed primarily of MCF10.DCIS cells. Reconstructed confocal image of MCF10.DCIS-RFP cells (red) grown in MAME co-culture with CAF40TKi-CFSE cells (green) for 8 days. CAF40TKi CFSE-labeled fibroblasts clustered near invasive edges (arrows). 20X magnification. ***P < 0.001. (TIFF 150 kb) [file 12885_2015_1576_MOESM5_ESM.tiff]

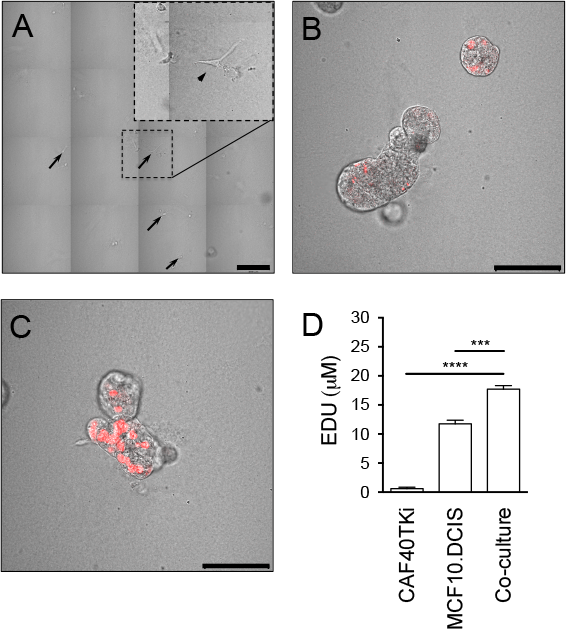

Supplement: Additional file 7: Figure S4. — MCF10.DCIS: CAF40TKi MAME co-culture increased cell proliferation. (A) Lack of 5-Ethynyl-2´-deoxyuridine (EDU) thymidine analog incorporation in CAF40TKi fibroblasts grown for 5 days. Arrows point out fibroblasts scattered throughout the culture. Scale bar, 200 μm. (B) Incorporation of EDU in MCF10.DCIS cells grown for 5 days. (C) Incorporation of EDU in a representative structure from a MCF10.DCIS: CAF40TKi co-culture. Note that similar sized structures were chosen to compare levels of EDU incorporation in a 24-h period. Scale bars, 100 μm. (D) DNA was extracted from MAME cultures to quantify EDU concentration. Data shows a significantly higher concentration of EDU incorporation in DNA of co-culture as compared to monotypic cultures. ***P < 0.001, ****P < 0.0001, Student’s t-test; mean ± SD. (TIFF 344 kb) [file 12885_2015_1576_MOESM7_ESM.tiff]

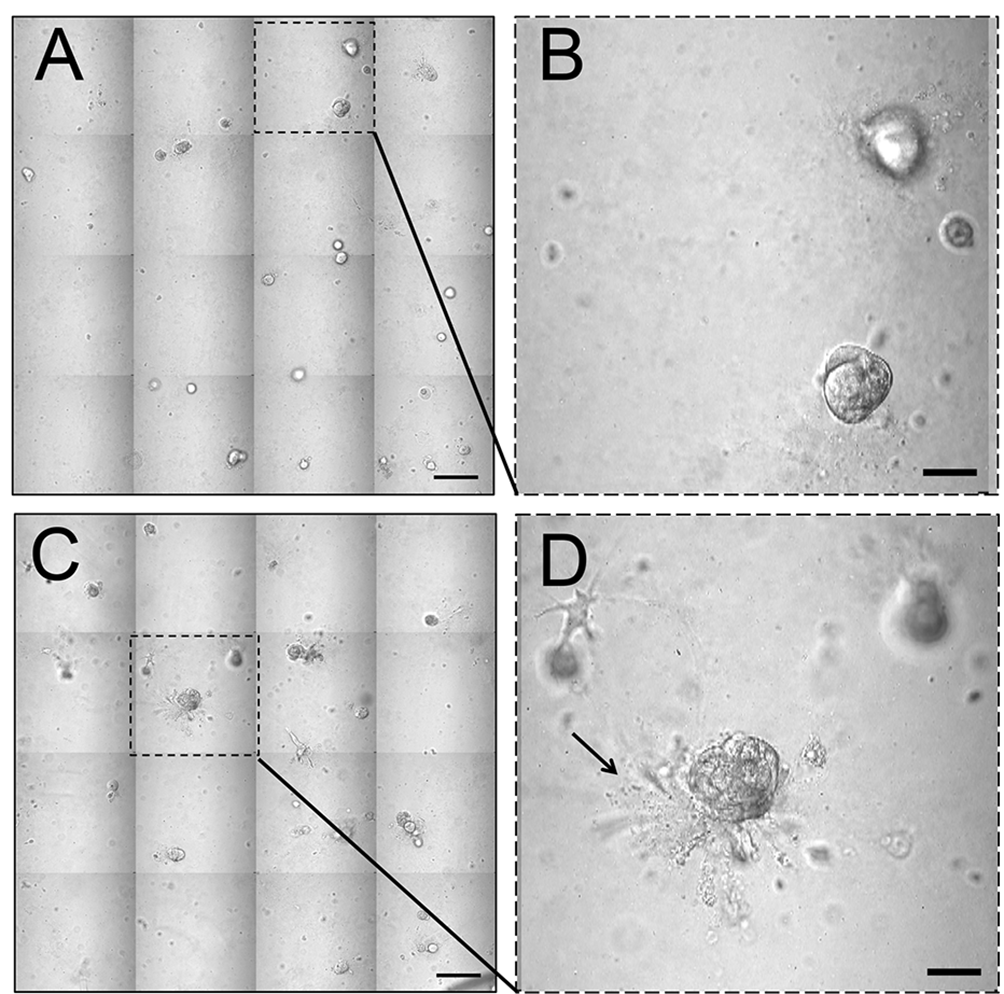

Supplement: Additional file 8: Figure S5. — Carcinoma-associated fibroblasts enhance invasive growth of SUM102 cells in MAME co-culture. (A) Representative contiguous tile image of SUM102 cells grown in MAME culture for 8 days (N = 3). (B) Single field from panel A shows spheroidal SUM102 structures. (C) Representative MAME co-culture of SUM102 and CAF40TKi cells (N = 3). (D) Single field from panel C shows SUM102 cells having many invasive outgrowths (arrow). Scale bar, 50 μm (B and D), 200 μm (A and C). (TIFF 963 kb) [file 12885_2015_1576_MOESM8_ESM.tiff]

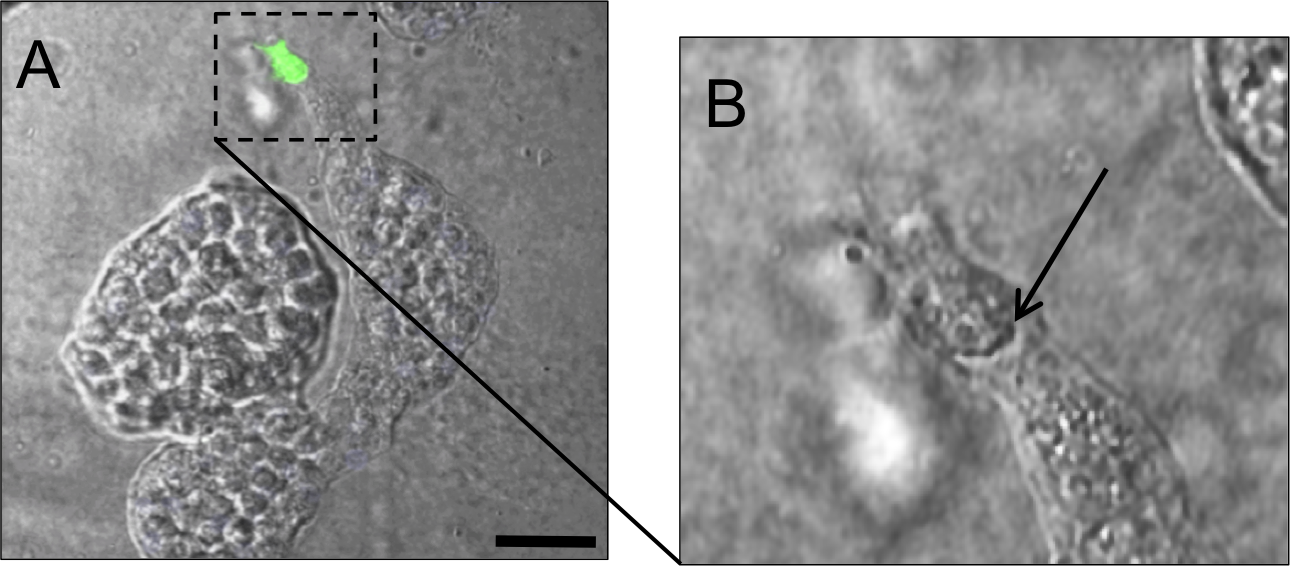

Supplement: Additional file 10: Figure S6. — MCF10.DCIS multicellular structures form heterocellular contacts with CAFs. (A) An 8-day 3D MAME co-culture of MCF10.DCIS cells and CAF40TKi fibroblasts show a CFSE labeled fibroblast at the invasive edge of a multicellular DCIS structure. Scale bar equals 50 μm. (B) High magnification zoom reveals the contact region as a dense demarcation between tumor and fibroblast. Color removed to enhance visualization of heterocellular contact. (TIFF 766 kb) [file 12885_2015_1576_MOESM10_ESM.tiff]

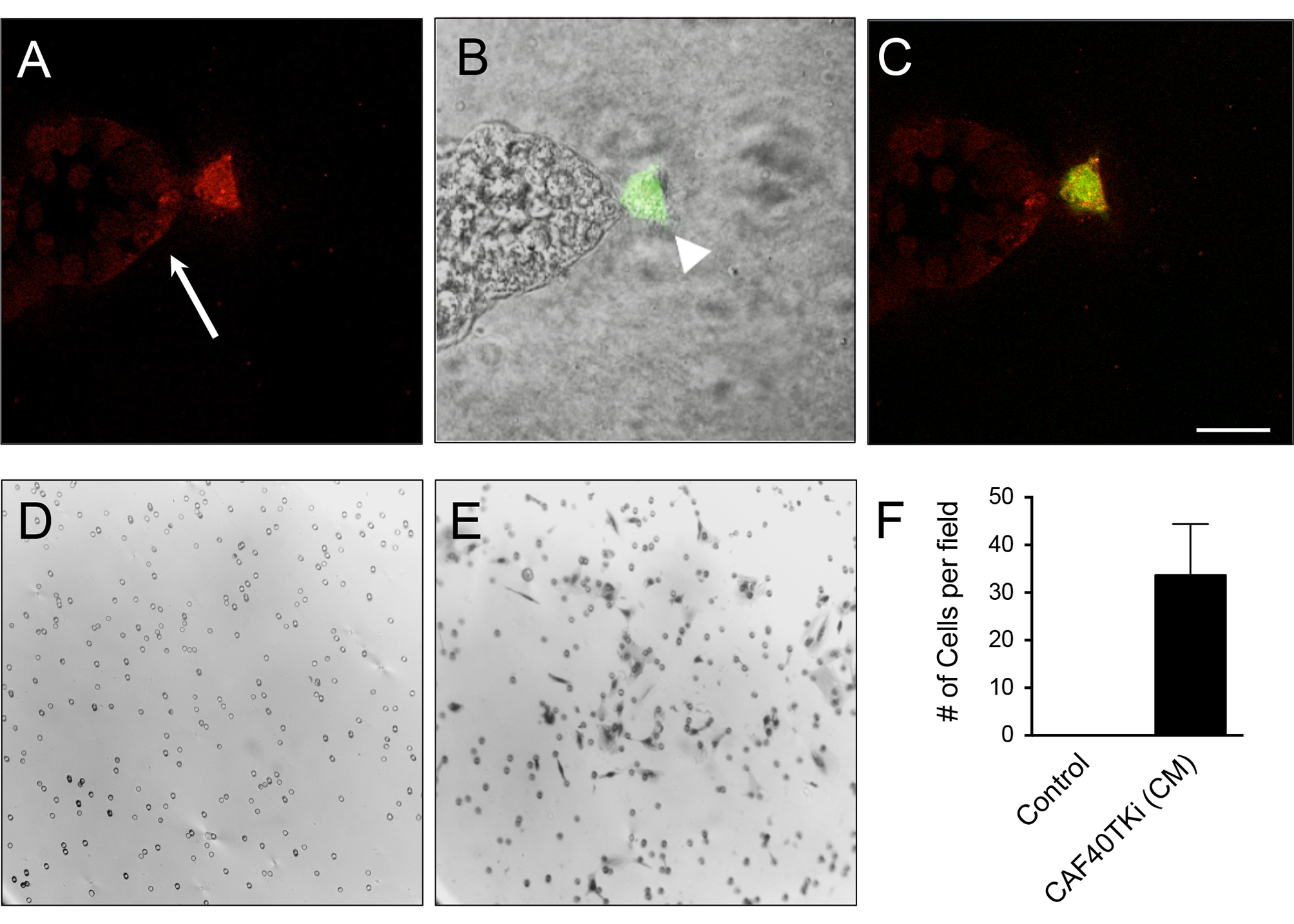

Supplement: Additional file 13: Figure S7. — CAF-conditioned medium induces MCF10.DCIS cell migration. (A) A representative CAF40TKi fibroblast expresses IL-6 (red) as it leads an MCF10.DCIS structure during collective migration (collective migration is inferred based on observations from time-lapse microscopy, see Movie 2). An MCF10.DCIS structure (arrow) shows a gradient of IL-6 expression (red) that is most intense near a cell:cell attachment with a CAF40TKi fibroblast. (B) DIC/fluorescent overlay image identifies the single leading cell as a CFSE-labeled CAF40TKi fibroblast (green). MCF10.DCIS cells are unlabeled. (C) Fluorescence only overlay of panels A and B. Scale bar, 20 μm. (D) A migration assay membrane showed that MCF10.DCIS cells do not migrate through transwell membrane pores to serum-free media. (E) MCF10.DCIS cell migrate through the transwell membrane pores to CAF40TKi conditioned serum-free media. (F) Quantification of visible cells that have migrated through transwell membrane filter after 24 h. (TIFF 11394 kb) [file 12885_2015_1576_MOESM13_ESM.tiff]

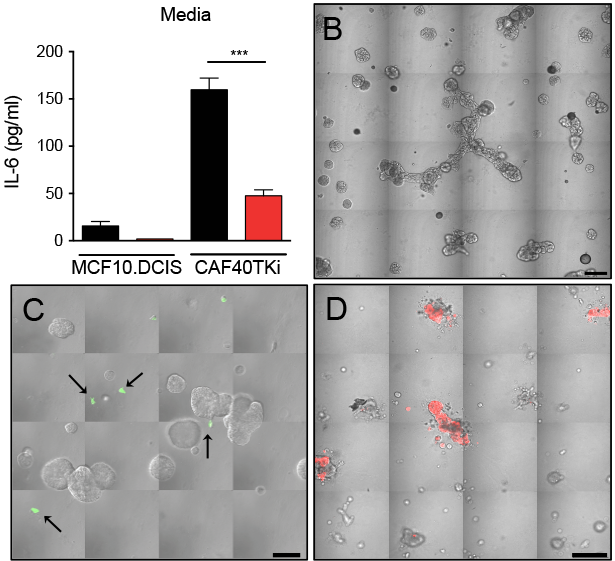

Supplement: Additional file 14: Figure S8. — Knockdown of IL-6 in CAF40TKi fibroblasts inhibits their interaction with MCF10.DCIS cells. (A) CAF40TKi fibroblasts and MCF10.DCIS cells were transduced with an shRNA targeting IL-6 mRNA. ELISA quantification of IL-6 secreted from control (black bars) and IL-6 targeted shRNA cell lines (red bars). (B) MCF10.DCIS cells co-cultured with CAF40TKi fibroblasts transduced with a scrambled shRNA virus. (C) Co-culture of MCF10.DCIS cells (unlabeled) with IL-6 shRNA transduced CAF40TKi fibroblasts (green). (D) Co-culture of IL-6 shRNA transduced MCF10.DCIS cells (red) with CAF40TKi fibroblasts (unlabeled). Scale bars, 200 μm. ***P < 0.001. All statistics are Student’s t-test; mean ± SD. (TIFF 319 kb) [file 12885_2015_1576_MOESM14_ESM.tiff]

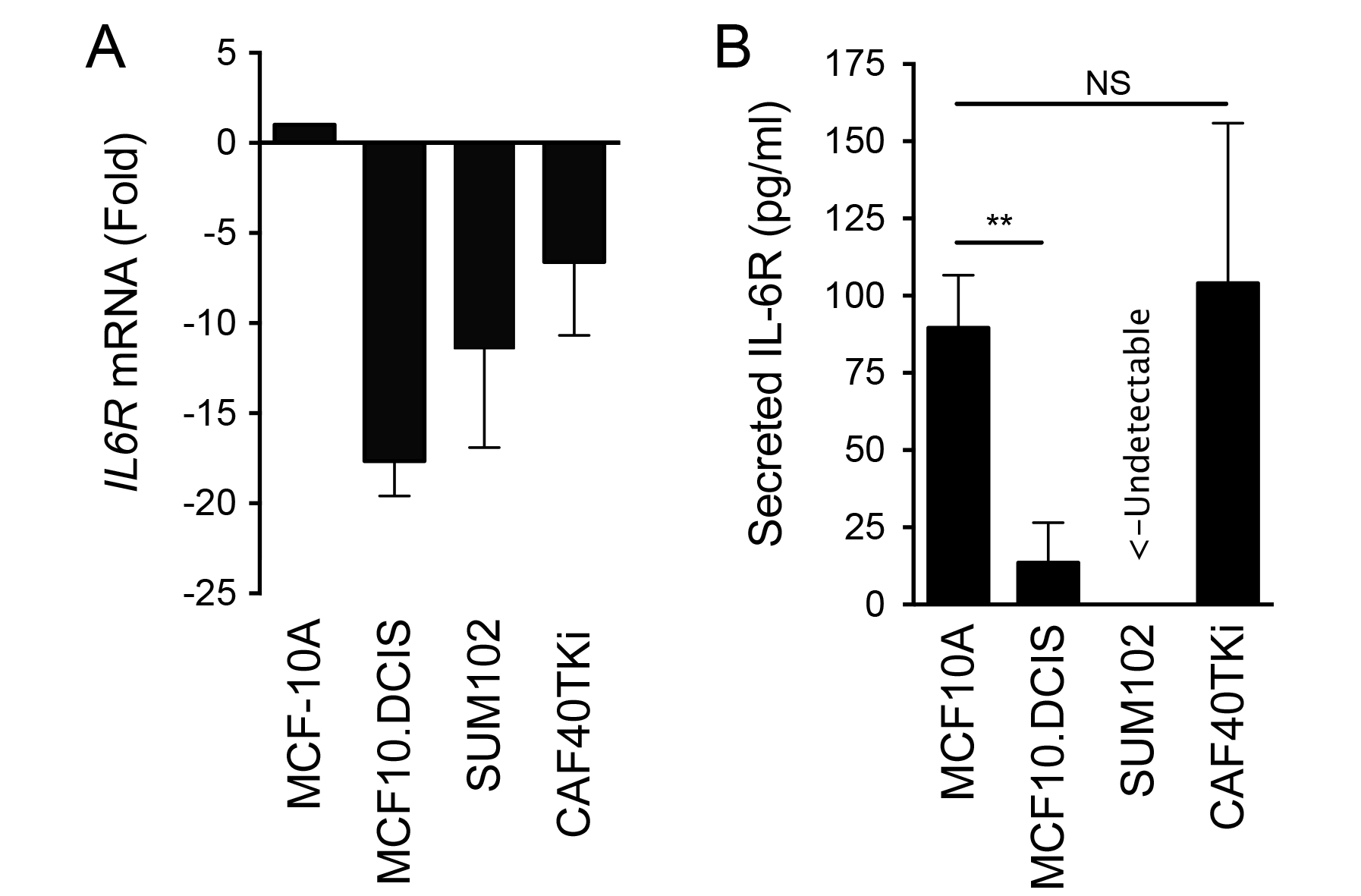

Supplement: Additional file 15: Figure S9. — IL-6 receptor gene expression and protein secretion in DCIS cell lines and CAF40TKi fibroblasts. (A) Examination of IL-6R gene expression. Fold difference as compared to MCF-10A non-tumor-forming breast epithelial cells. (B) Measurement of soluble IL-6R in media collected from MAME cultures (determined by ELISA). **P ≤ 0.001, NS. Not significant, by Student’s t-test; mean ± SD. (TIFF 119 kb) [file 12885_2015_1576_MOESM15_ESM.tiff]
